# Production of Active Poly- and Oligosaccharidic Fractions from *Ulva* sp. by Combining Enzyme-Assisted Extraction (EAE) and Depolymerization

**DOI:** 10.3390/metabo9090182

**Published:** 2019-09-12

**Authors:** Mathilde Fournière, Thomas Latire, Marie Lang, Nolwenn Terme, Nathalie Bourgougnon, Gilles Bedoux

**Affiliations:** 1 Université Catholique de l’Ouest Bretagne Nord, 22200 Guingamp, France; tlatire@uco.fr; 2 Laboratoire de Biotechnologie et Chimie Marines, EA 3884 Université Bretagne Sud, 56000 Vannes, France; marie.lang@univ-ubs.fr (M.L.); nolwenn.terme@univ-ubs.fr (N.T.); nathalie.bourgougnon@univ-ubs.fr (N.B.); gilles.bedoux@univ-ubs.fr (G.B.)

**Keywords:** green seaweed, polysaccharide, ulvan, enzyme-assisted extraction, endo-protease Protamex^®^, depolymerization, human dermal fibroblast, lipoxygenase

## Abstract

Data on fractionation and depolymerization of the matrix ulvan polysaccharides, and studies on the biological activities on skin cells, are very scarce. In this work, crude ulvans were produced by using EAE (enzyme-assisted extraction) and compared to maceration (an established procedure). After different fractionation procedures—ethanolic precipitation, dialysis, or ammonium sulfate precipitation—the biochemical composition showed that EAE led to an increased content in ulvans. Coupling EAE to sulfate ammonium precipitation led to protein enrichment. Oligosaccharides were obtained by using radical depolymerization by H_2_O_2_ and ion-exchange resin depolymerization. Sulfate groups were partially cleaved during these chemical treatments. The potential bioactivity of the fractions was assessed using a lipoxygenase inhibition assay for anti-inflammatory activity and a WST-1 assay for human dermal fibroblast viability and proliferation. All ulvans extracts, poly- and oligosaccharidic fractions from EAE, expanded the fibroblast proliferation rate up to 62%. Our research emphasizes the potential use of poly- and oligosaccharidic fractions of *Ulva* sp. for further development in cosmetic applications.

## 1. Introduction

Green seaweed from the *Ulva* genus represents an important biomass of the European coasts due to its proliferation. Algal blooms due to marine eutrophication have a detrimental impact on the environment and local economy, especially in Brittany, France [1,2]. For now, *Ulva* sp. is less up-graded in Brittany and used as soil amendment and animal feed or is simply degraded by combustion or by letting them rot [1]. Seaweeds are sources of high value-added products with potential for the natural cosmetic industry [3,4,5,6,7,8]. The possible use of green seaweeds as an important and abundant source of cosmeceutical bioactive compounds has emerged in recent years [9].

Interest in extraction, fractionation, and purification of high value compounds from *Ulva* sp. has risen significantly for the exploitation of this natural resource. The cell wall of *Ulva* sp. is composed of several polysaccharide types constituting the skeleton—celluloses, hemicelluloses, mannans, and xylans—and the matrix phase—ulvans, xyloarabinogalactans, and glucuronoxylorhamnans [10,11]. Water-soluble sulfated polysaccharides, known as ulvans, are mainly composed of sulfated rhamnose, uronic acids (glucuronic and iduronic), and xylose [12]. The two main repeating disaccharide units of ulvans are aldobiuronic acids, referred to ulvanobiuronic acids: A_3_S—β-d-glucuronic acid (1,4)-linked to α-l-rhamnose 3-sulfate—and B_3_S—α-l-iduronic acid (1,4)-linked to α-l-rhamnose 3-sulfate [11,13]. Traditionally, the extraction of ulvans is performed by hot water extraction, also called maceration, in the presence of chelators or acids used to disrupt the cell wall, and followed by ethanolic precipitation [13]. Nowadays, a novel technology called EAE (enzyme-assisted extraction) is employed to improve the extraction of polysaccharides in terms of yield, time, and cost and to reduce energy consumption [14,15].

*Ulva* sp., especially its ulvans, have demonstrated in tubo, in vitro and in vivo biological activities such as immunomodulation, antioxidant, anticancer, anticoagulant, antihyperlipidemic, or anti-viral [8,13,16,17,18,19,20,21]. The structural feature of ulvans corresponding to its molecular weight, degree of sulfation, sulfation pattern, monosaccharide composition, glycosidic linkages, isomers, and degree of branching influences its biological activity [13]. Few studies have reported the effect of *Ulva* sp. compounds on skin cells. Fibroblasts are the main cells from the papillary dermis, lying directly under the epidermis. They have the principal function of maintaining the integrity of ECM (extracellular matrix) and its regulation, through the synthesis of type I collagen but also proteoglycans and glycosaminoglycans [22,23,24]. Fibroblasts have the capacity to regenerate the skin and are involved in skin aging (including extrinsic and intrinsic) [25,26]. Lipoxygenases are enzymes implicated in inflammation processes through the synthesis of leukotrienes and participate therefore in the secretion of reactive oxygen species (ROS). Oxidative stress, including ROS production, is known to play a major role in skin aging by decreasing collagen synthesis by fibroblasts and increasing the synthesis of MMP (matrix metalloproteases, enzymes implicated in the ECM catabolic pathway with collagen degradation) [27].

The first aim of this study was to produce poly- and oligosaccharidic fractions from the green seaweed *Ulva* sp. by comparing a classical extraction process (maceration) and green technology EAE using endo-protease Protamex^®^, followed by depolymerization. The different fractions were characterized by their biochemical composition and molecular weight distribution. The second aim of the study was to investigate the in tubo and in vitro biological activities of these fractions rich in poly- and oligosaccharides. Lipoxygenase inhibition and human dermal fibroblast viability and proliferation were studied in order to highlight the potential of fractions rich in poly- and oligosaccharides from *Ulva* sp. in skin care.

## 2. Results

### 2.1. Extraction and Fractionation of Ulvans

#### 2.1.1. Yields of Extraction and Fractionation

**Figure 1:**
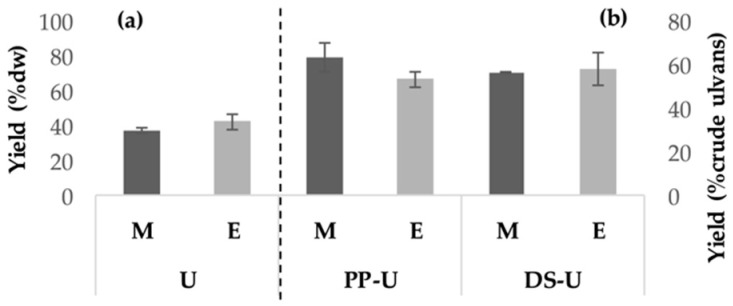
Yields of (**a**) crude ulvans and (**b**) fraction production after enzyme-assisted extraction or maceration. Crude ulvans (U) or the fractions PP-U (after second ethanolic precipitation) and DS-U (after dialysis) were obtained from maceration (M) or enzyme-assisted extraction (E). Crude ulvans yields were expressed as a percentage of seaweed dry weight (% dw) and fractions yields as a percentage of recovery from crude ulvans (% crude ulvans).

Detailed processes of the production of crude ulvans and fractions are shown in Figure 6 (Section 4.1.1). Figure 1 shows yields of extraction of crude ulvans (U) and fractions production (PP-U and DS-U) after enzyme-assisted extraction or maceration.

For U and DS-U, EAE led to better yields than maceration but not significantly, with 42.5 ± 4.4% dw and 58.4 ± 7.3% crude ulvans, respectively (*p* < 0.05). However, second ethanolic precipitation (PP-U) after EAE led to lower yield (53.5 ± 3.3% crude ulvans) but not significantly (*p* < 0.05) than maceration. PP-U and DS-U production led to similar yields. Yields of production of AS-DS-U and AS-DP-U were 38.0 and 4.3% crude ulvans, respectively.

#### 2.1.2. Biochemical Characterization of Ulvans

Table 1 shows the biochemical composition of crude ulvans (U) and fractions called high-molecular-weight polysaccharides (HMWPs, including PP-U, DS-U, AS-DS-U, and AS-DP-U) in mineral matter, carbohydrates, uronic acids, sulfate groups, and proteins expressed in percentage of dry weight (% dw). U, PP-U, DS-U, and AS-DS-U fractions were rich in ulvans according to their great content in carbohydrates (21.9–37.4%), uronic acids (18.5–37.0%), and sulfate groups (25.9–49.4%).

Fractionation using the three procedures (PP-U, DS-U, AS-DS-U, and AS-DP-U) led to a significant (*p* < 0.05) enrichment in carbohydrates from crude ulvans U (from +7% up to +14%). The carbohydrate contents were the highest in dialyzed fractions DS-U, AS-DS-U, and AS-DP-U (31.5–37.4%). Uronic acids were significantly (*p* < 0.05) present in a larger proportion in PP-U, DS-U, and AS-DS-U (23.7–37.0%), when compared to crude ulvans U (18.5%). AS-DP-U fractions were significantly (*p* < 0.05) poorer in uronic acids (13.3%) than AS-DS-U (36%). Sulfate group contents were significantly (*p* < 0.05) superior in PP-U, DS-U, and AS-DS-U fractions, ranging from 41.9 to 49.4% compared to crude ulvans U (29.9%) and weaker in AS-DP-U fractions (23.9%). U, PP-U, DS-U, and AS-DS-U fractions exhibited similar content in proteins (8.9–12.8%). However, the most abundant protein content was observed in AS-DP-U fractions (16.4%) and enriched significantly (*p* < 0.05) from crude ulvans U (+6%). Maximum concentrations of mineral matter were detected in crude ulvans U (35.2%). PP-U exhibited a significant decrease of 7% in mineral matter content. The lowest mineral content was observed in DS-U (13.4%) thanks to the salt elimination during the dialysis process.

When compared to maceration, EAE led to a significant (*p* < 0.05) rise in carbohydrates (for PP-U and AS-DS-U), in uronic acids (for DS-U and AS-DS-U), and in sulfate groups for all fractions (see Table S1a).

Table 2 indicates the monosaccharide composition of crude ulvans (U) and HMWP fractions (PP-U, DS-U, AS-DS-U, and AS-DP-U). Values represent the mean of percentage of the different monosaccharides, relative to the total carbohydrate content (g/100 g total carbohydrates). Fructose, glucosamine, ribose, mannose, and arabinose were poorly (< 1%) or not detected (data not shown). Iduronic was not analyzed due to the absence of a standard. Rhamnose (29.1–59.0%), glucose (4.0–14.0%), and glucuronic acid (3.4–11.9%) are the most representative monosaccharides. Rhamnose represented almost half of the carbohydrate content for U, PP-U, DS-U, and AS-DS-U (with a maximum of 59%). Both ethanolic precipitation and dialysis led to a significant (*p* < 0.05) decrease in glucose (−3 to −6%) from crude ulvans U. Glucuronic acid represented for almost all fractions 10% of the monosaccharide content, except for AS-DP-U with 3.4%. Xylose was poorly detected with amounts ranging from 2.7 to 4.3%. The same observation was made for galactose ranging from 1.3 to 1.6% (for U, PP-U, DS-U, and AS-DS-U) up to 6.2% (AS-DP-U).

Minor significant differences were observed between maceration and EAE for monosaccharide composition and were most likely related to glucose content (see Table S2a).

### 2.2. Oligosaccharide Production by Ulvans Depolymerization

#### 2.2.1. Monitoring of Depolymerization by High Performance Size Exclusion Chromatography (HPSEC)

**Figure A1:**
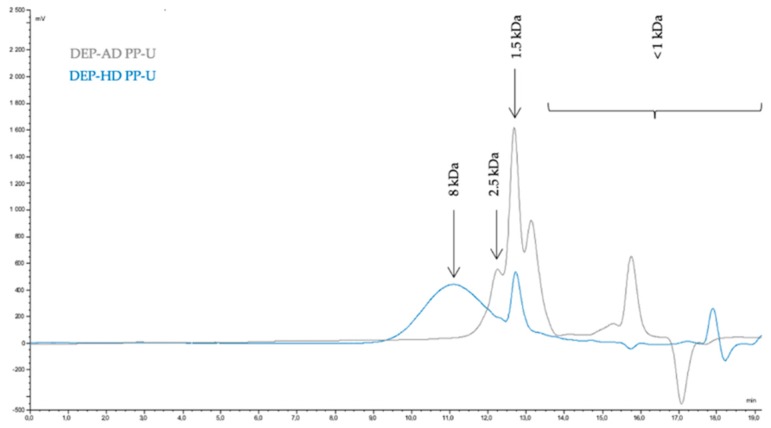
High pressure steric exclusion chromatogram on G3000PW_XL_ column (RI) of depolymerized fractions DEP-AD PP-U and DEP-HD PP-U from EAE. Flow rate: 0.7 mL/min; eluent: 0.1 M NaNO_3_; sample: 1 mg/mL; detection: refractive index (RI).

Fractions of oligosaccharides are called low-molecular-weight polysaccharides (LMWPs). The molecular weight distribution of polysaccharides has been determined by HPSEC (Figure A1) and by using dextran standards.

Two depolymerization processes were applied on PP-U (HMWPs of approximately 3000 kDa) and were effective. Resin depolymerization with Amberlite according to an optimized protocol of Adrien et al. (2017), 24 h at 80 °C, led to a low molecular weight of 1.5 kDa (M_n_ = 1.5 ± 0.06 kDa and M_w_ = 1.6 ± 0.08 kDa) with a low polydispersity index of 1.1, indicating a homogeneous size profile of the fraction. No differences were observed between DEP-AD PP-U from maceration and EAE. Depolymerization with hydrogen peroxide used in the work of Pengzhan et al. (2004) was optimized to see the influence of time and temperature on depolymerization. Optimization was done after 24 h at 50 °C and led to the formation of DEP-HD PP-U with an average M_w_ of 8 kDa. Depolymerization increased with temperature and time (Table 3).

#### 2.2.2. Yields of Oligosaccharide Production

Yields of depolymerization were expressed in the percentage of recovery from PP-U (% PP-U). No significant differences in yield were observed between maceration and EAE (*p* < 0.05) for DEP-AD PP-U (34.3 ± 9.2% from EAE). The yield of DEP-HD PP-U (55.7%) was greater than DEP-AD PP-U.

#### 2.2.3. Biochemical Characterization of Oligosaccharides

Table 4 presents the biochemical composition of LMWP fractions in carbohydrates, uronic acids, sulfate groups, and proteins expressed in a percentage of dry weight (% dw). LMWP fractions were rich in carbohydrates and uronic acids. Significant (*p* < 0.05) larger proportions of carbohydrates and uronic acids in DEP-AD PP-U were observed (+6 and +9%, respectively).

The depolymerization process with ion-exchange resin led to a partial loss of sulfates linked to the polysaccharide backbone. Indeed, with the Azure A method, no sulfate was detected in these fractions. H_2_O_2_ depolymerization led to partial cleavage of sulfate groups (around 6%). Proteins were abundant in both depolymerized fractions (12.8–16.8%).

Table 5 indicates the monosaccharide composition of LMWP fractions. Values represent the mean of the percentage of different monosaccharides relative to the total carbohydrate content (g/100 g total carbohydrates). Fructose, glucosamine, ribose, mannose, and arabinose were poorly (< 1%) or not detected (data not shown). Rhamnose (44.9–55.4%), glucuronic acid (7.5–11.0%), and glucose (4.9–7%) were abundant in depolymerized fractions. Rhamnose represented almost half of the carbohydrate content. The maximum glucuronic acid content was found in DEP-AD PP-U and significantly (*p* < 0.05) differed from DEP-HD PP-U. However, low contents in xylose (2.1–2.6%) and galactose (1.0–1.7%) were detected.

No significant differences in monosaccharide composition were observed between depolymerized fractions from EAE and maceration (Table S2b).

#### 2.2.4. Matrix-Assisted Laser Desorption Ionization-Time of Flight (MALDI-TOF) Mass Spectrometry

**Figure 2:**
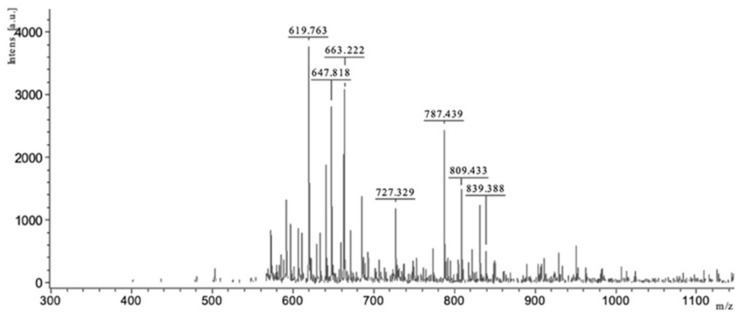
MALDI-TOF-mass spectrum of DEP-AD PP-U from *Ulva* sp.

MALDI-TOF mass spectrum of DEP-AD PP-U from EAE (Figure 2) could reveal the presence of several oligosaccharides. Labels for interpretation are as follows: Glc: Glucose; Xyl: Xylose; Rha: Rhamnose; GlcA: Glucuronic acid.

On the basis of their molecular weight, pseudo-molecular ions at *m/z* 839, 809, 737, 663, 647, and 619 might correspond to disulfated Glc-Xyl-Rha-GlcA, disulfated Glc-Xyl-(Rha)_2_, monosulfated Glc-Xyl-Rha-GlcA, disulfated Glc-Xyl-Rha, disulfated Xyl-(Rha)_2_, and Xyl-(Rha)_2_-GlcA, respectively. The monosaccharides detected by HPAEC-PAD (see Section 2.2.3) were confirmed by MALDI-TOF.

### 2.3. Biological Activities

#### 2.3.1. Lipoxygenase Inhibition Assay by Crude Ulvans and HMWP and LMWP Fractions from *Ulva* sp.

**Figure 3:**
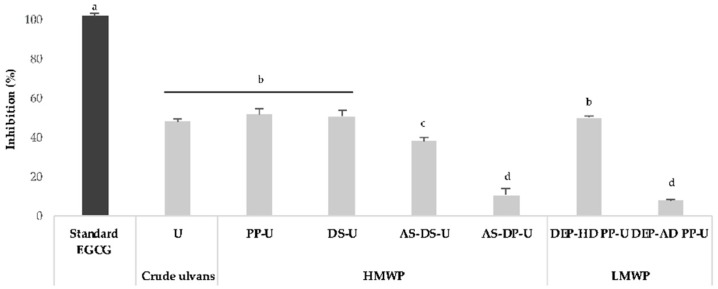
Effect of *Ulva* sp. extracts and fractions on lipoxygenase inhibition. Crude ulvans and HMWP and LMWP fractions were obtained from EAE. EGCG (Epigallocatechin gallate) was used as a standard. The data are presented as the means ± SE of triplicate runs. Letters when different represent significant differences according to Tukey’s pairwise a posteriori test after ANOVA, considering *p* < 0.05.

Extracts and fractions from *Ulva* sp. were screened for anti-inflammatory activity using the soybean lipoxygenase inhibition technique (Figure 3).

EGCG (Epigallocatechin gallate) as a positive control at 250 μg/mL was effective with 100% inhibition of lipoxygenase. Lipoxygenase inhibition ranged from 48 to 52% with U, DS-U, AS-DS-U, and DEP-HD PP-U at 500 μg/mL. A significant (*p* < 0.05) lower inhibition was observed with AS-DP-U and DEP-AD PP-U fractions (8 and 10%, respectively). It appeared that lipoxygenase inhibition was not altered by an enriched polysaccharide fraction obtained after ammonium sulfate precipitation (AS-DS-U with 38% inhibition). H_2_O_2_ depolymerization significantly (*p* < 0.05) raised the inhibitory activity of the fractions when compared to depolymerization by ion-exchange resin.

Significant differences (*p* < 0.05) in activity between maceration and EAE were observed for PP-U, AS-DS-U, and AS-DP-U (+11, −11, and −12%, respectively) (see Figure S1).

#### 2.3.2. Modulation of Fibroblast Proliferation by Crude Ulvans and HMWP and LMWP Fractions from *Ulva* sp.

**Figure 4:**
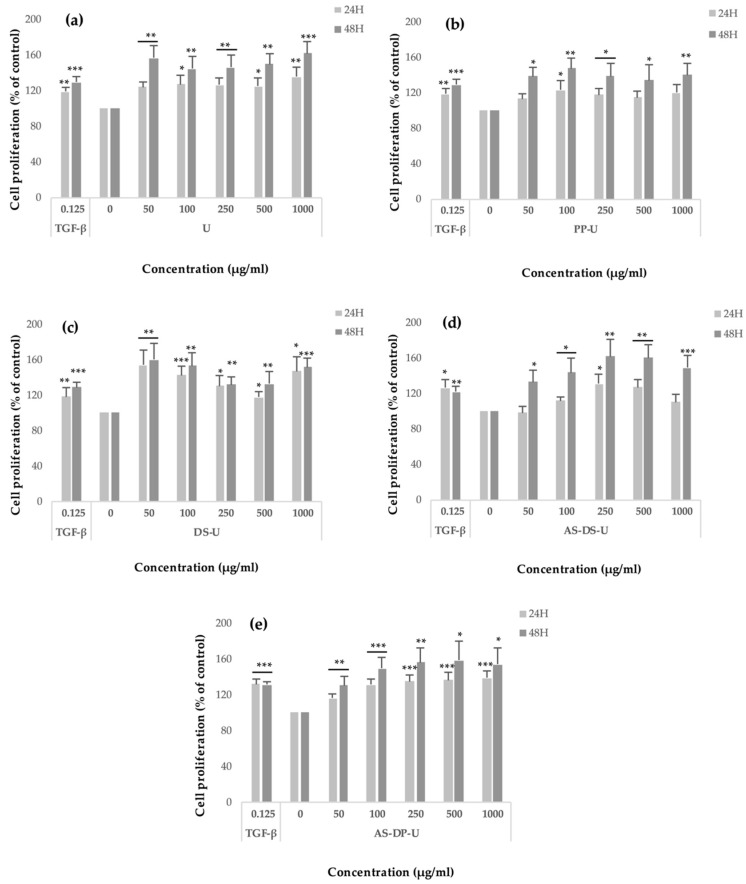
Effect of polysaccharidic extracts and fractions from *Ulva* sp. from EAE on fibroblast proliferation evaluated by the WST-1 assay after incubating cells in the presence of extracts (50–1000 μg/mL) for 24 and 48 h. Effects of (**a**) U, (**b**) PP-U, (**c**) DS-U, (**d**) AS-DS-U and (**e**) AS-DP-U from EAE were evaluated. TGF-β1 was used as positive control. Significant differences compared with negative control (0) according to a Student’s *t*-test are indicated by asterisks (* *p* < 0.05, ** *p* < 0.01, *** *p* < 0.001), *n* = 6.

**Figure 5:**
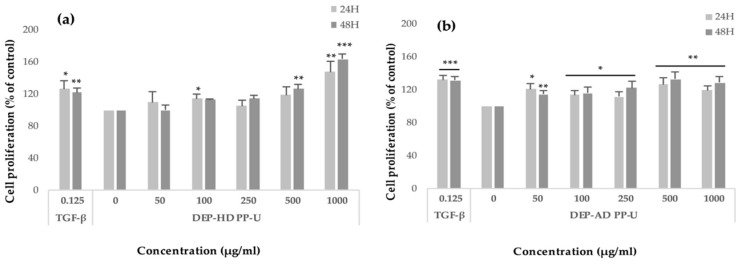
Effect of depolymerized fractions from *Ulva* sp. from EAE on fibroblast proliferation evaluated by the WST-1 assay after incubating cells in the presence of extracts (50–1000 μg/mL) for 24 h and 48 h. Effects of (**a**) DEP- HD PP-U and (**b**) DEP-AD PP-U from EAE were evaluated. TGF - β1 was used as positive control. Significant differences compared with negative control (0) according to a Student’s *t*-test are indicated by asterisks (* *p* < 0.05, ** *p* < 0.01, *** *p* < 0.001), *n* = 6.

**Figure 6:**
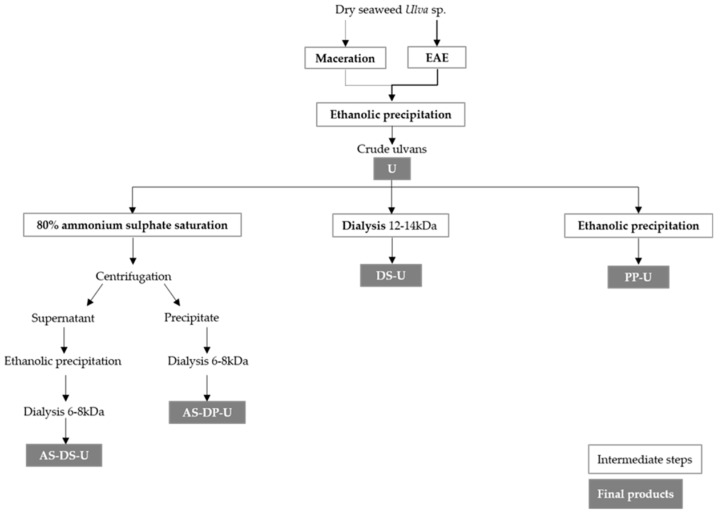
Procedure of extraction and fractionation of ulvans from *Ulva* sp.

Crude ulvans and HMWP and LMWP fractions from EAE were evaluated on human dermal fibroblast (HDF) proliferation using the WST-1 assay (Figure 4 and Figure 5). HDF were treated for 24 h and 48 h with 50, 100, 250, 500, and 1000 μg/mL of the different fractions.

When compared to untreated cells, a significant increase (i.e., statistically significant: *p* < 0.05 or less) in the proliferation was observed in the presence of crude ulvans (Figure 4a) from +24 to +35% after 24 h of incubation and from +45 to +62% after 48 h of incubation. Maximum significant proliferation (*p* < 0.001) was observed at concentration 1000 μg/mL after 48 h of incubation (+62%).

For PP-U (Figure 4b), almost non-significant proliferation (i.e., not statistically significant or NS) was measured from +13 to +23% after 24 h of incubation except for PP-U at 100 μg/mL where it was significant (*p* < 0.05). After 48 h of incubation, fibroblast proliferation was significant from +34 to +47%.

Dialyzed fractions DS-U (Figure 4c) increased proliferation significantly after 24 and 48 h of incubation up to +60%. The maximum proliferation was reached at 50 μg/mL (+54% after 24 h and +60% after 48 h of incubation, *p* < 0.01). A significant augmentation (*p* < 0.001) of cell proliferation was also observed at 1000 μg/mL for DS-U after 48 h of incubation (+51%).

Figure 4d shows that, after 48 h of incubation, AS-DS-U had a significant effect on the proliferation rate of fibroblasts up to +62% at 250 and 500 μg/mL (*p* < 0.01). After 24 h of incubation, proliferation was lower from 0 to 34% and not always significant. The most significant increases (*p* < 0.001) were reached after 48 h of incubation at 1000 μg/mL (+49%).

AS-DP-U fractions (Figure 4e) boosted also significantly proliferation after 24 and 48 h of incubation (from +16 up to +58%). The cell proliferation rose significantly with concentrations in a relative dose-dependent manner.

All crude ulvans and HMWP fractions increased fibroblast proliferation up to 62%. Observations show that, after 48 h of incubation, significant fibroblast proliferation rate was greater than after 24 h.

Figure 5a shows that, after 48 h of incubation, DEP-HD PP-U increased the cellular proliferation in a dose-dependent manner. The maximum significant rates (*p* < 0.001) were reached at concentrations of 500 and 1000 μg/mL after 48 h of incubation (+26%, *p* < 0.01 and +63%, *p* < 0.001, respectively).

For DEP-AD PP-U (Figure 5b), all concentrations enhanced fibroblast proliferation significantly. Maximum proliferations were significantly reached after 48 h of incubation at 500 and 1000 μg/mL (+28 and +32%, *p* < 0.01).

Similar biological activity was observed for crude ulvans and fractions produced from maceration (see Figures S2 and S3).

## 3. Discussion

### 3.1. Extraction and Fractionation of Ulvans

#### 3.1.1. Ulvan Production

In our study, we compared two extraction processes: maceration (conventional technique) and EAE followed by fractionation procedures. EAE is considered an environmentally friendly technique of extraction [15,28].

Yields of extraction and fractionation of ulvans from maceration and EAE ranged from 7.4 to 42.5% dw. A review of Kidgell et al. (2019) indicated that ulvan extraction yields ranged from 0.1 to 90% dw [13]. Another review of Lahaye et al. (2007) presented yields of ulvan extraction between 8 and 29% dw [12]. Our results are in accordance with these data. Indeed, it is well known that the quantitative yield of ulvan extraction could vary depending on the extraction and purification processes but also between populations of *Ulva* due to eco-physiological variation (temperature and light) [13].

In our study, EAE led to higher yields than maceration (for crude ulvans, dialyzed fractions, and dialyzed ammonium sulfate precipitation supernatant), albeit sometimes not significant. Larger yields with EAE are in agreement with a previous study of Hardouin et al. (2016), where the endo-protease Protamex^®^ was used to produce enzymatic aqueous extracts from *Ulva* sp. with a yield of 88% with EAE and 44% with maceration [17]. A previous study using EAE on *Ulva* spp. with cellulase (Celluclast^®^) and protease (Alcalase^®^) treatments followed by ethanol precipitation allowed the recovery of a weaker yield with 12.2% polysaccharides [29]. EAE enhanced the solubilization and fragmentation of cell wall components [28].

In our study, the production of PP-U from maceration led to a yield of 24% dw. Furthermore, similar extraction using maceration for the production of U and PP-U was performed by Hardouin et al. (2015) and led to lower yields of 24 and 8.2% dw, respectively [30].

DS-U yields ranged from 19.6 to 21.4% dw for maceration and EAE, respectively. From studies of Lahaye et al. (1998; 1999) and Thanh et al. (2016), ulvan recovery yields, after the first ethanol precipitation and dialysis, were between 8 and 15% dw [31,32,33], which is weaker than our results.

#### 3.1.2. Biochemical Characterization of Ulvans

It is noteworthy that different biochemical compositions were identified in the fractions. Fractionation procedures from crude ulvans (PP-U, DS-U, and AS-DS-U) led to their purification. Crude ulvans, PP-U, DS-U, and AS-DS-U were mainly composed of rhamnose, glucuronic acid, xylose, and sulfates, which represent the main constituents of ulvans [34,35]. Ulvans were most abundant in dialyzed fractions (DS-U and AS-DS-U). Work of Lahaye et al. (1999) on several *Ulva* samples exhibited contents in rhamnose (27.9–58.3%), glucose (5.0–38.1%), galactose (0.9–3.1%), xylose (5.4–28.8%), and glucuronic acid (11.6–30.4%) [31]. In the review of Kidgell et al. (2019), the following content values were reported: rhamnose: 5.0–92.2 mol %; glucuronic acid: 2.6–52.0 mol %; xylose: 0.0–38.0 mol % [13]. Our results are in the range of a previous analysis of ulvans from *Ulva* species [12,13,31].

Glucose and galactose quantified in extracts are often reported in the literature as an unclear component or contaminant of ulvans [13]. In our study, glucose appeared to be a contaminant of ulvans due to its decrease in PP-U and dialyzed fractions while ulvans are enriched.

Despite little variation of yield between maceration and EAE, EAE increased contents in sulfates and carbohydrates, specific components of matrix polysaccharides from *Ulva* sp. Crude ulvans from EAE using Protamex^®^ led to similarities with Celluclast^®^ and Alcalase^®^ extraction for uronic acids (18.5 against 19.3 mol %), xylose (3.2% for both), and galactose (1.6 against 1.3%) but also to differences in rhamnose (50.4 against 28.2%) and glucose (14.0 against 5.9%) [29]. It is interesting to note that the protease Protamex^®^ led to a larger enrichment in carbohydrates (rhamnose and glucose in particular) than cellulase (Celluclast^®^) and protease (Alcalase^®^).

Mineral matter content of crude ulvans was characterized by high values (35.2% dw) and reduced with ethanolic precipitation and with dialysis cut-off of 12–14 kDa. Mineral matter content of crude ulvans is in accordance with previous studies of Yaich et al. (2013) (33.4–44.1%) and Costa et al. (2012) (43.2%) [36,37]. Dialysis range of MW cut-off used for ulvans was generally between 3.6 and 12 kDa [13]. The dialysis process of crude ulvans (a drop of 25% of ash content) efficiently declines mineral matter and enriches the fraction in ulvans.

An analysis of the fractions revealed the presence of proteins. A purification step using ethanolic precipitation from crude ulvans (10.5% dw) failed to lower the protein content (11.2% dw for PP-U). Sulfated polysaccharides are closely associated with proteins within the cell wall structure as previously described [38], and they confirmed the protein content of our fractions.

Ammonium sulfate precipitation is traditionally used directly after the aqueous extraction of seaweed for protein extraction [39,40,41]. Our fractionation procedure from crude ulvans using ammonium sulfate led to a 1.6-fold increase in the protein proportion. Fraction AS-DP-U, enriched in carbohydrates (31.5%) and proteins (16.4%), and reduced in uronic acids and sulfates, suggest a decreased of co-precipitated ulvans during the process. Protein content was, however, significantly lower than in previous literature (76.3% dw from *Ulva lactuca*) [42]. The biochemical composition of AS-DP-U could be related with the branching between sulfated and branched polysaccharides associated with various proteins [43]. Cell wall polysaccharides could disturb the extraction and purification of proteins [42,44]. In the work of Harrysson et al. (2018), *Ulva lactuca* protein separation using ammonium sulfate led to extracts with lower protein content than crude seaweed. They also found that this traditional method concentrates carbohydrates from the raw material [39]. Even if in our study the protein content of AS-DP-U was significantly higher than the respective crude ulvans, a significantly increased concentration of carbohydrates was also observed. To our knowledge, this is the first study relating the use of ammonium sulfate precipitation to crude ulvans.

### 3.2. Depolymerization of Ulvans for Oligosaccharide Production

#### 3.2.1. Oligosaccharide Production

PP-U fractions exhibited the high molecular weights of ulvans, and those from maceration (M_n_ = 1691 kDa and M_w_ = 3171 kDa) were larger than those from EAE (M_n_ = 1651 kDa and M_w_ = 2290 kDa). Differences in molecular weights could be linked to the temperature of extraction (90 °C for maceration against 50 °C for EAE), revealing that elevated temperature was suitable for extraction of high-molecular-weight ulvans [12]. EAE could also improve the hydrolysis of cell wall components resulting in lower macromolecular polydispersity (1.4 against 1.9). Fractions were in the range 530–7700 kDa of the molecular weights of ulvans, based on the work of Lahaye et al. (2007), and in the range 1–2200 kDa, based on a review by Kidgell et al. (2019) [12,13].

Several depolymerization processes can be applied to polysaccharides such as chemical depolymerization (acid-alkali hydrolysis, radical depolymerization, or H_2_O_2_-induced depolymerization [9,18,32,45]), enzymatic hydrolysis (using ulvan-lyase or glucuronan-lyase for example [46,47]), and physical depolymerization (thermal, microwave, γ-irradiation, and ultrasonication) [48,49]. In our study, we selected only chemical procedures with acid hydrolysis using ion-exchange resin Amberlite FPC23 H and H_2_O_2_-induced depolymerization to produce LMWPs followed by dialysis in order to purify the fraction. Depolymerization by a radical process using H_2_O_2_-generated hydroxyl free radicals (a powerful oxidant) and led to the formation of radical carbon [49,50]. Hydrogen peroxide can be considered as a clear reagent due to its decomposition into oxygen and water [45].

In this study, ulvan depolymerization using ion-exchange resin led to a yield of 7.9% dw. Hydrogen peroxide depolymerization yielded 56.4 and 55.7% dw from maceration and EAE, respectively. The DEP-AD PP-U yield is lower than the yield of 10.5% dw from the study of Lahaye et al. (1998) using acid IR 120 resin [32], but DEP-HD PP-U yields are in accordance with the results of Pengzhan et al. (2004) (58.4–67.7% dw) depending on their degradation conditions [45].

The depolymerization of PP-U using Amberlite (DEP-AD PP-U) was effective and allowed for the production of lower molecular weights (1.5 kDa). Good repeatability of resin depolymerization and the production of homogeneous samples (polydispersity index of 1.1) presented advantages compared to acid treatment using hydrochloric, sulfuric, trifluoroacetic, formic, or nitrous acids, which are hard to control and led to the production of monosaccharides and adverse products [49]. Acidic depolymerization of ulvans by mean of resins for 24 h at 80 °C has already been reported [9,32]. The combination of elevated temperature (80 °C), low pH, and a long extraction duration (24 h) led to significant depolymerization [13]. It is suggested that the acid hydrolysis of polysaccharides is realized by the cleavage of *O-*glycosidic linkage occurring with nucleophile substitution reaction SnI [49]. The molecular weight of DEP-AD PP-U is in accordance with a previous study of Adrien et al. (2017), in which the same depolymerization process led to a relatively higher average molecular weight of 4 kDa [9]. The lower average molecular weight obtained in our study could be related to the constant contact of resin to the polysaccharide solution.

Optimization of depolymerization from undegraded ulvans PP-U using H_2_O_2_ was performed. The longer time of depolymerization (24 h) led to degraded ulvans DEP-HD PP-U of low molecular weight (8 kDa). However, weak repeatability of DEP-HD PP-U production from maceration was observed. Previous works have been done on ulvan depolymerization using H_2_O_2_ but with maximum parameters assessed of 7 h and 50 °C [18,45]. Low-molecular-weight ulvans can be effectively prepared by H_2_O_2_ treatment, and the rate of depolymerization is dependent on the reaction time and temperature [18,45,51]. However, free-radical depolymerization is suggested to be random and could explain the relatively weak repeatability of oligosaccharide production [49]. Qi et al. (2005) confirmed that the H_2_O_2_ depolymerization process broke glycosidic linkages without affecting the basic chemical structure of ulvans [18].

#### 3.2.2. Biochemical Characterization of Oligosaccharides

LMWP fractions exhibited substantial contents in carbohydrates (with rhamnose, glucose, and xylose) and uronic acids (with glucuronic acid). DEP-AD PP-U was significantly enriched in carbohydrates and uronic acids, and weaker in sulfate groups and proteins, relative to DEP-HD PP-U. Sulfate contents were decreased (0–6.6% dw detected by the Azure A method). However, the pseudo-molecular ions identified by MALDI-TOF of DEP-AD PP-U suggested the presence of sulfate groups linked to the polysaccharidic chain. H_2_O_2_ depolymerization allowed for the retaining of 5.0–6.6% dw of sulfates. The polysaccharidic chain of ulvans occurred in these fractions [12] but without sulfate group bonding. The depolymerization caused a substantial desulfation of ulvans despite degradation under mild conditions to prevent it. In previous studies, desulfation was a side reaction of the depolymerization method [11,34]. However, resin depolymerization performed by Adrien et al. (2017) allowed for the retaining of 70% of sulfates (whether 8% dw) [9], which differs from our study.

Further investigations will be done using ^13^C NMR to evaluate desulfation and elucidate oligosaccharide structures.

It is noteworthy that DEP-AD PP-U from EAE exhibited significant superior protein content than maceration. The use of the endo-protease Protamex^®^ could explain the augmented protein extraction yields [28].

### 3.3. Biological Activities

#### 3.3.1. Anti-Inflammatory Activity by Lipoxygenase Inhibition

Lipoxygenases (LOX) are monomeric proteins, containing dioxygenase catalyzing the regio- and stereo-specific dioxygenation of PUFA (polyunsaturated fatty acids) containing a (1Z, 4Z)-pentadiene system such as linoleic, linolenic, and arachidonic acids, and their esters into hydroperoxides [52,53,54]. Inhibition of LOX by EGCG has been reviewed by Chedea et al. (2011) [52]. Due to its action on PUFA, lipoxygenase enzyme is known to play a key role in inflammation [52,55].

In our study, all crude ulvans and HMWP and LMWP fractions exhibited anti-inflammatory activity as determined by LOX inhibition. Crude ulvans, DS-U, PP-U, and DEP-HD PP-U showed the strongest antioxidant activity of LOX inhibition. Several studies in the literature reported the antioxidant and anti-inflammatory abilities of seaweeds by lipoxygenase inhibition [56,57,58,59,60,61,62]. Two different works presented lower results of LOX inhibition by aqueous extract of green seaweeds *Enteromorpha linza* (around 9%) [56] and *Ulva lactuca* (24%) [59]. Other studies on *Ulva lactuca* presented anti-inflammatory and antioxidant activities of methanol extracts enriched in carbohydrates [58,59]. Fucose-containing sulfated polysaccharides (previously fucoidans) from *Padina tetrastromatica* also decreased LOX activity and showed anti-inflammatory activity [57].

The enzymatic addition of oxygen to fatty acid could be one of the targets of ulvans, resulting in the inhibition of the activity [56]. Ion-exchange resin depolymerization with desulfated lowest molecular weight led to a reduction in inhibition activity. Therefore, the present study suggests that the anti-inflammatory activity was related to fractions rich in poly- and oligosaccharides.

#### 3.3.2. Effects of the Ulvans Extracts and Fractions on Fibroblast Proliferation and Viability

The present study showed fibroblast proliferation and cell viability when incubated with ulvans extracts and poly- and oligosaccharidic fractions enriched in rhamnose, with concentrations ranging from 50 to 1000 μg/mL. Our results are in accordance with previous studies revealing that ulvans were largely non-cytotoxic on different cell types (macrophage cell lines [63,64,65], gut cells [66], fibroblast cells from mouse [67], and Vero cells [68]).

The results revealed that crude ulvans and HMWP fractions produced from EAE increased fibroblast proliferation up to 62%. Oligosaccharides (DEP-AD PP-U < 2 kDa and DEP-HD PP-U < 9 kDa) induced an increase of fibroblast proliferation (up to +63%). In the literature, studies have already demonstrated that *Ulva* compounds affect the metabolism or fibroblast proliferation maintained in vitro [9,69,70,71]. In particular, experiments performed on human dermal fibroblasts demonstrated that hydrolyzed *Ulva pertusa* extracts induced an increase in proliferation rate of the cells after incubation (+30% at 250 μg/mL) [70]. In the same way, Ennamany et al. (1998) have shown that human skin fibroblasts proliferated significantly when incubated with SECMA 1^®^, a mitogenic hexapeptide from *Ulva* sp. [69]. A study of Andrès et al. (2006) revealed that rhamnose-rich poly- (45 and 50 kDa) and oligosaccharides (5 and 14.5 kDa), with 50–60% rhamnose, boosted fibroblast proliferation from 40 to 80% [72]. Several studies suggested that the α-l-rhamnose was detected by lectin-site on human skin fibroblasts [72,73]. However, Adrien et al. (2017) showed that fibroblast proliferation was decreased when incubated with ulvan extracts and depolymerized ulvans (by ion-exchange Amberlite resin) [9]. Rioux et al. (2013) showed that radical depolymerized galactofucans from brown seaweed (< 10 kDa) at 1000 μg/mL enhanced fibroblast proliferation compared to a negative control [74]. Therefore, extraction and depolymerization procedures of polysaccharides have a significant effect on the biological activities of extracts. Overall, the results obtained in our work with crude ulvans and fractions from *Ulva* sp. have biological activities in agreement with most of the literature data.

Crude ulvans and HMWP and LMWP fractions stimulated fibroblast cell proliferation with no relevant cytotoxicity. This result highlights the potential of crude ulvans and *Ulva* sp. fractions for safe use. According to our knowledge, this study shows for the first time the increase of fibroblast proliferation and viability with oligosaccharides from *Ulva* sp.

## 4. Materials and Methods

### 4.1. Extraction and Fractionation Procedure (Figure 6)

The green macroalgae *Ulva* sp. (Chlorophyta, Ulvales, Ulvaceae) was collected on the beach Landrézac (47°30′17.9”N 2°42′37.1”O) in Sarzeau (Brittany, France) on 28 May, 2018. Seaweeds were then washed with tap water, ground to a 3 mm diameter, frozen at −25 °C, and freeze-dried (Alpha 1-4 LSC, Christ).

#### 4.1.1. Enzyme-Assisted Extraction (EAE) Followed by Polysaccharide Precipitation

Dry material of *Ulva* sp. underwent eco-extraction, referred as enzyme-assisted extraction (EAE), and was compared to a classic method known as maceration.

For EAE, enzyme endo-protease Protamex^®^ (Novozymes, Bagsværd, Denmark) (6%, w/dw) was added to 800 mL of distilled water previously heated at 50 °C. Afterwards, 33 g of dry algal matter were added. Enzymatic hydrolysis was performed at 50 °C for 3 h under continuous stirring at 300–500 rpm. EAE was stopped by inactivating the enzyme for 15 min at 90 °C (denaturation) [17]. For maceration, 800 mL of distilled water were heated at 90 °C, and dry algal matter was added (33 g) in the bioreactor (Mac Technologie, Fontenay-Trésigny, France). The mixture was heated for 2 h at 90 °C under continuous stirring at 300–500 rpm [12]. After extraction, the samples were filtered and pressed on a Büchner system using a cheesecloth to remove algal residues.

Aqueous extracts obtained were submitted to ethanolic precipitation with absolute ethanol (1:5, *v/v*, Fisher Chemical) at 4 °C for 24 h. After Büchner filtration, the precipitates were freeze-dried and stored at 4 °C. Extracts are named U for crude ulvans and were produced in triplicates.

#### 4.1.2. Fractionation of Crude Ulvans

Three different procedures of fractionation were applied to crude ulvans.

I. Ethanolic precipitation

Crude ulvans (10 mg/mL in distilled water) were submitted to ethanolic precipitation (1:5, *v/v*) at 4 °C for 24 h. After Büchner filtration, the precipitates were freeze-dried and stored at 4 °C. These fractions were named PP-U (polysaccharide precipitation) and were produced in quadruplicates.

II. The dialysis process

Crude ulvans (10 mg/mL in distilled water) were dialyzed against distilled water for seven days (cut-off 12–14 kDa, Spectra/Por®4 Dialysis Membrane, Spectrum Laboratories) by replacing the water twice every 24 h for the three first days and once every 24 h for the next four days. The content was centrifuged at 5000 g for 15 min at 4 °C (Avanti J-30 I Centrifuge, Beckman). The supernatants were freeze-dried, stored at 4 °C, and named DS-U (dialyzed supernatant). DS-U fractions were produced in duplicates.

III. Polysaccharide precipitation following ammonium sulfate precipitation

A first ammonium sulfate precipitation was applied to crude ulvans in order to recover proteins [75]. One gram of extracts was dissolved in 34 mL of phosphate buffer, 20 mM, pH 7, at 4 °C for 24 h under continuous stirring, and ammonium sulfate (4 mol/L, 80% saturation) was added. Solutions were stirred overnight at 4 °C and then centrifuged at 4000 *g* at 4 °C for 1 h.

Pellets were dissolved in distilled water, dialyzed against distilled water for 72 h at 4 °C (cut-off 6–8 kDa, Spectra/Por^®^1) by replacing the water twice every 24 h, freeze-dried, and stored at 4 °C. These fractions are referred to as AS-DP-U (dialyzed precipitate after ammonium sulfate precipitation) and were produced only once.

Supernatants were collected for polysaccharide precipitation with absolute ethanol (1:5, *v/v*) at 4 °C for 2 h under stirring. Solutions were centrifuged at 10,000 g for 15 min at 4 °C. Pellets were dissolved in distilled water, dialyzed against distilled water for 72 h at 4 °C (cut-off 6–8 kDa) by replacing the water twice each 24 h, freeze-dried, and stored at 4 °C. These fractions are referred to as AS-DS-U (dialyzed supernatant after ammonium sulfate precipitation) and were produced only once.

### 4.2. Production of Oligosaccharides by Ulvan Depolymerization

**Figure 7:**
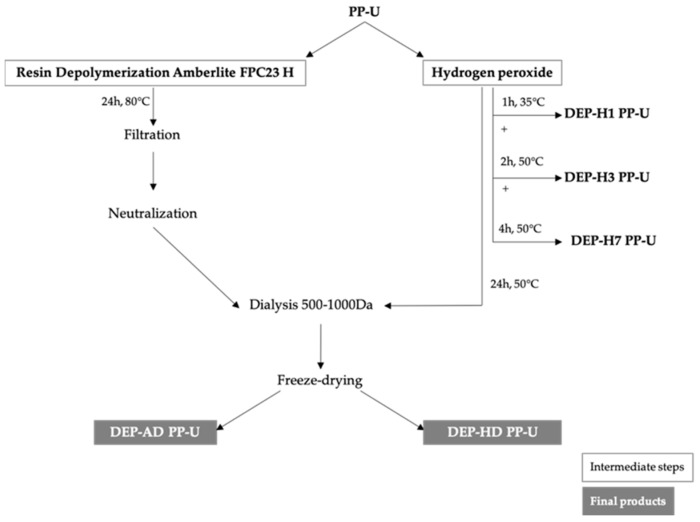
Detailed processes of depolymerization using resin Amberlite FPC23 H and H_2_O_2._

Depolymerization was applied only to PP-U using two protocols (Figure 7): H_2_O_2_ depolymerization and ion-exchange resin depolymerization.

Depolymerization was followed by high-performance gel permeation chromatography.

#### 4.2.1. Hydrogen Peroxide Depolymerization

Hydrogen peroxide (100 volumes >30%, Fisher Scientific) was used for depolymerization of polysaccharide solutions [18,45]. Polysaccharide solution PP-U was prepared at 25 mg/mL in distilled water. Hydrogen peroxide was added to the solution (8%, *v/v*), and the mix was heated under stirring (35 or 50 °C). Aliquots were collected at different time and temperature of experiments (Table 6). Fractions DEP-HD PP-U were dialyzed 48 h against distilled water (cut-off 500–1000 Da, Biotech CE Tubing, Spectra/Por^®^) by replacing the water twice each 24 h, freeze-dried, and stored at 4 °C. All others fractions were directly freeze-dried and stored at 4 °C. These fractions are named DEP-HD PP-U (depolymerized by H_2_O_2_ and dialyzed from PP-U) and were produced only once.

#### 4.2.2. Depolymerization by Ion-Exchange Resin

Resin Amberlite^®^ FPC23 H (Sigma-Aldrich), a strong acidic resin, was used for the depolymerization of polysaccharides [9]. Polysaccharide solution of PP-U (100 mL at 25 mg/mL) was homogenized by Ultraturax (Polytron PT 31000 D). Amberlite resin (10 mL) was added to the solution. Depolymerization was performed at 80 °C under stirring for 24 h. After cooling down, the solution was filtered and neutralized with 1 and 0.1 M NaOH.

Fractions were dialyzed for 48 h against distilled water (cut-off of 500–1000 Da, Biotech CE Tubing, Spectra/Por^®^) by replacing the water twice each 24 h, freeze-dried, and stored at 4 °C. These fractions are referred to DEP-AD PP-U (depolymerized by Amberlite and dialyzed from PP-U) and were produced in triplicates.

### 4.3. Polysaccharide Molecular Weight (MW) Distribution by HPSEC Analysis

Mass analyses of extracts enriched in ulvans and depolymerized fractions were determined on UHPLC Ultimate 3000 (Thermo Scientific) using guard column PW_XL_ and size-exclusion chromatography columns: TSK gel G6000PW_XL_ (30 cm × 7.8 mm, 13 μm, MW range 5 × 10^5^–5 × 10^7^ Da), G4000PW_XL_ (30 cm × 7.8 mm, 10 μm, MW range 1 × 10^3^–7 × 10^5^ Da) or G3000PW_XL_ (30 cm × 7.8 mm, 6 μm, MW range < 6 × 10^4^ Da); see Table 7 for details. The analysis temperature was stabilized at 30 °C. Elution was performed with a 0.1 M sodium nitrate (NaNO_3_, Fisher Chemical) solution at 0.7 mL/min. Poly- and oligosaccharides were detected by differential refractometry (Iota 2, Precision Instrument), and chromatograms were analyzed by Chromeleon software. Samples were prepared at 1 mg/mL in an eluent and filtered at 0.45 μm (Millex-HV, Merck). The standard curve was made using dextran standards with a molecular weight ranging from 1000 to 670,000 Da (Sigma-Aldrich). Number-averaged molecular weight (M_n_), weight-averaged molecular weight (M_w_), and polydispersity index (I) were calculated as follows [76]:

(1) $$M_{n}{=}_{\;}\frac{\left(\sum N_{i}\;\times {\;M}_{i}\right)}{\sum N_{i}\;}$$

(2) $$M_{w}{=}_{\;}\frac{\left(\sum N_{i}\;\times {\;M}_{i}{}^{2}\right)}{\left(\sum N_{i}\;\times {\;M}_{i}\right)}$$

(3) $$I=\frac{M_{w}}{M_{n}}$$

where N_i_ was the number of moles of the polysaccharide species, and M_i_ the molecular weight.

### 4.4. Biochemical Composition Analysis

Mineral matter content was determined by a calcination method with measurement of mass loss of samples after drying for 40 min at 100 °C, burning with a Bunsen burner (5 min), and calcining 2 h at 585 °C.

Prior to analysis, dried samples (10 mg) were hydrolyzed for 2 h at 100 °C with 1 M hydrochloric acid (5 mL) and neutralized with 1 M sodium hydroxide (5 mL) (Fisher Chemical) for uronic acid, carbohydrate, and protein quantifications. For sulfate group quantification, dried samples (10 mg) were hydrolyzed for 2 h at 100 °C with ultrapure water (10 mL).

Carbohydrate content was determined according to a phenol sulfuric method [77] using glucose as standard. Uronic acid content was measured using sulfamate/m-hydroxydiphenyl assay and glucuronic acid as standard [78]. Protein content was determined using a BCA kit assay (bicinchoninic acid) and bovine serum albumin as a standard [79]. Sulfate content was measured using Azure A (3-amino-7-(dimethylamino) phenothizin-5-ium chloride), binding to sulfated groups in a polysaccharide chain, and dextran sulfate as a standard [80].

### 4.5. Monosaccharide Composition Determined by High Performance Anion Exchange Chromatography

Monosaccharide analyses of extracts and fractions were performed using high performance anion exchange chromatography with pulsed amperometric detection (HPAEC-PAD, Dionex ICS-5000 + DC). Prior to analysis, 1 mL of sample (2 mg/mL) was hydrolyzed for 48 h at 100 °C with 110 μL of 1 M hydrochloric acid in sealed glass vials. After hydrolysis, samples were neutralized with 110 μL of 1 M sodium hydroxide. For calibration, monosaccharide standards, rhamnose, glucosamine, galactose, mannose, fructose, arabinose, glucose, xylose, ribose, deoxyribose, and glucuronic acid were purchased from Fisher Chemical, Sigma-Aldrich and Acros Organics. Deoxyribose was used as an internal standard in each sample at 50 ppm. Twenty-five microliters of the sample were injected into an analytical column (CarboPac PA1, 4 × 250 mm) equipped with a guard column (CarboPac Guard Column, 4 × 60 mm). Elution was done at a 1 mL/min flow rate by three eluents of 0.1 M sodium hydroxide (NaOH), 1 M sodium acetate (NaOAc) in 0.1 M NaOH, and ultrapure water. The system was maintained in isocratic mode 18% 0.1 M NaOH/82% H_2_O for the first 20 min. A NaOAc gradient from 0 to 100% was then applied at the 20th minute for 10 min and was kept isocratic at 100% for 5 min. Finally, the system returned to initial isocratic conditions (18% 0.1 M NaOH/82% H_2_O) for an additional 45 min. Identification and quantification of monosaccharides were performed using Chromeleon software.

### 4.6. Matrix-Assisted Laser Desorption Ionisation-Time of Flight (MALDI-TOF) Mass Spectrometry

Samples of DEP-AD PP-U (2 μL at 1 mg/mL in HPLC water, VWR Chemicals) were mixed with 2 μL of the matrix solution (2,5-dihydroxybenzoic acid 10 mg/mL in TFA:CH_3_CN:1.75:0.75; *v/v*). A total of 1 μL of this solution was applied to a stainless steel sample slide and dried [81]. MALDI-TOF mass spectra of the generated oligomers were recorded on a MALDI-TOF Microflex (Bruker) mass spectrometer. Spectra were acquired in the reflectron mode. Dextran standards of 1 and 5 kDa (Sigma-Aldrich) were used as a calibration mixture for the MALDI-TOF analysis.

### 4.7. Biological Activities

#### 4.7.1. Lipoxygenase Inhibition Assay

For this in tubo assay, in 96-well microplates, 90 μL of a solution of soybean lipoxygenase from *Glycine max* type I-B (Merck) at 560 U/mL in a borate buffer (0.2 M pH = 9, Thermo Fisher Scientific) were distributed in each well (enzyme’s final concentration per well: 252 U/mL). Fifty microliters of the samples (final concentration per well: 500 μg/mL) were added and completed with 50 μL of the borate buffer. Fifty microliters of (-)-Epigallocatechin gallate, 95%, (Acros Organics) were used as a positive control (final concentration per well 250 μg/mL). The positive control and samples were prepared in ultrapure water and filtered on a 0.20 μm cellulose acetate membrane. The plate was sealed and incubated at 25 °C for 10 min. Ten microliters of linoleic acid (1 mM emulsified in ultrapure water: ethanol, 9:1, *v/v*, Merck) were then added to each well. A first absorbance reading was performed with a microplate reader at 234 nm, and successive readings were performed for 10 min at 25 °C to assess the percentage of inhibition.

Inhibition activity was calculated from the slope obtained from the regression line by the following formula:

(4) $$I\;\left(\%\right)=\left(\frac{\mathrm{Ablank}\;–\;\mathrm{Asample}\;}{\mathrm{Ablank}}\right)\times 100$$

where A_blank_ is the slope obtained for the enzyme in the presence of ultrapure water, and A_sample_ is the slope obtained for the enzyme in the presence of inhibitor/sample. Triplicate measurements were performed.

#### 4.7.2. Cell Culture

Human dermal fibroblasts samples were provided by Laboratoire Interactions Epithéliums Neurones (LIEN, EA 4685), Brest, France. Human dermal samples were obtained from skin biopsies of healthy donors undergoing abdominoplasty surgery. All patients signed an informed consent agreement form. The study was conducted in accordance with the Declaration of Helsinki. Sample collections adhered to the local agreement comity (Comité de protection des personnes Ouest VI) and referenced under DC 2016-2833.

Skin samples were adhered in a 25 cm² culture flask coated with type I collagen (Sigma-Aldrich) and Dulbecco’s modified eagle medium (DMEM, Gibco) with 10% (*v/v*) fetal bovine serum (FBS, Gibco), a 1% antibiotic solution (*v/v*) (Penicillin, Streptomycin 10000 U/mL, Gibco), and 1% antifungal (*v/v*) (Fungizone, amphotericin B, Gibco) were added. Fibroblasts were spread on a culture flask for 10 days, and skin explants were removed afterwards. Cells were cultured in a temperature-controlled incubator with 5% CO_2_ at 37 °C.

Cells were subcultured by trypsinization (0.05%) and EDTA solution (Gibco) after reaching confluence. All experiments were performed between the 3rd and 8th passages.

For assays, cells were seeded onto 96-well microplates at a density of 4000 cells/well. After reaching 80% confluency, cells were incubated in DMEM with 2% FBS in the absence or presence of samples for 24 h and 48 h (50, 100, 250, 500, and 1000 μg/mL).

#### 4.7.3. WST-1 Assay

The WST-1 in vitro assay is based on the conversion of tetrazolium salt WST-1 (4-[3-(4-Iodophenyl)-2-(4-nitrophenyl)-2H-5-tetrazolio]-1,3-benzene disulfonate) into formazan (orange dye) by cellular mitochondrial deshydrogenases. The color change is directly proportional to the viability and proliferation of cells in the culture. For the WST-1 assay, human TGF-β1 (Transforming Growth Factor-beta 1, Invitrogen) at 0.125 μg/mL was used as a positive control.

After incubation, the cell culture medium was removed, and 100 μL of the WST-1 reagent were added and incubated for 45 min at 37 °C with 5% CO_2_ (WST-1 cell proliferation kit, Roche Diagnostics, Meylan, France; 1:40 dilution in DMEM 2% FBS). Absorbance was measured at 450 and 630 nm using a microplate reader (Varioskan Lux, Thermo Scientific). The WST-1 assay was performed on six different skin cell patients.

### 4.8. Statistical Analysis

All data were presented as mean ± standard error. Statistical analyses were performed using Addinsoft 2018 and XLSTAT 2018: Data Analysis and Statistical Software for Microsoft Excel, Paris, France (2018). Biochemical composition and monosaccharide composition statistical analyses were done by a parametric ANOVA followed by Tukey’s pairwise a posteriori test at *p* < 0.05. Student’s *t*-test (independent, two-sided) was applied to the WST-1 results to determine significant differences between experimental and negative control samples.

## 5. Conclusions

This study focused on the production of ulvan extracts and fractions from *Ulva* sp. using different processes for the production of HMWPs: ethanolic precipitation, dialysis, and, for the first time, ammonium sulfate precipitation followed by ethanolic precipitation. Depolymerization of ulvans with hydrogen peroxide or ion-exchange resin produced LMWPs. HMWP and LMWP fractions had different compositions (enrichment in ulvans) and molecular weights (poly- and oligosaccharides). This study demonstrated that poly- and oligosaccharides enriched fractions have effects on anti-inflammatory activity and on human dermal cell proliferation. These results do not allow for the discrimination of different extracts and fractions according to any biological activity. Here, biological activity could not be related with a specific composition or a particular molecular weight. Indeed, it is interesting to note that the low molecular weights obtained after depolymerization retained a biological activity similar to that of the less purified extracts. Several studies have already shown that the molecular weight of compounds was related to the presence of a biological activity [74,82,83] and that those with the lowest molecular weight, especially polysaccharides, were potentially the best candidates [82,84].

This study suggests that bioactive compounds from *Ulva* sp. could be interesting for cosmetic applications especially in skin anti-ageing strategies.

However, further investigations are needed to identify the bioactive compounds (ulvans, proteins and/or poly- and oligosaccharides) and their in vitro mechanisms of action, considering different biological pathways (anabolic or catabolic with extracellular matrix synthesis or degradation) to adapt the findings for cosmetic use.

## Figures and Tables

**Table 1:** Biochemical composition (% dw) of crude ulvans and high-molecular-weight polysaccharides (HMWPs) from EAE.

	Mineral Matter	Carbohydrates	Uronic Acids	Sulfate Groups	Proteins
**Crude Ulvans**					
U	35.2 ± 0.2 ^a^	23.5 ± 0.5 ^c^	18.5 ± 0.6 ^c^	29.9 ± 0.5 ^c^	10.5 ± 0.4 ^b,c^
**HMWPs**					
PP-U	26.8 ± 1.0 ^b^	30.7 ± 0.2 ^b^	23.7 ± 0.7 ^b^	41.9 ± 0.5 ^b^	11.2 ± 0.2 ^b,c^
DS-U	13.4 ± 0.8 ^c^	37.4 ± 0.2 ^a^	37.0 ± 0.7 ^a^	49.1 ± 1.0 ^a^	12.8 ± 0.2 ^a,b^
AS-DS-U	-	35.1 ± 0.4 ^a,b^	36.0 ± 0.8 ^a^	49.4 ± 0.5 ^a^	8.9 ± 0.5 ^c^
AS-DP-U	-	31.5 ± 0.6 ^b^	13.3 ± 0.4 ^c^	23.9 ± 0.5 ^d^	16.4 ± 0.2 ^a^

“–“: not determined. Values are the mean ± SE (standard error). Different letters in the same column represent significant differences according to Tukey’s pairwise a posteriori test after ANOVA, considering *p* < 0.05.

**Table 2:** Monosaccharide composition (% carbohydrates) of crude ulvans and HMWPs from EAE.

	Rhamnose	Galactose	Glucose	Xylose	Glucuronic Acid
**Crude Ulvans**					
U	50.4 ± 6.0 ^a^	1.6 ± 0.1 ^b^	14.0 ± 1.2 ^a^	3.2 ± 0.2 ^a,b^	10.6 ± 0.9 ^a^
**HMWPs**					
PP-U	42.4 ± 0.3 ^a^	1.3 ± 0.1 ^b^	5.7 ± 0.7 ^b,c^	2.7 ± 0.2 ^b^	11.6 ± 1.9 ^a^
DS-U	50.2 ± 2.2 ^a^	1.6 ± 0.0 ^b^	3.3 ± 0.2 ^c^	2.8 ± 0.2 ^a,b^	11.9 ± 0.3 ^a^
AS-DS-U	59.0 ^a^	1.3 ^b^	4.0 ^b,c^	3.0 ^a,b^	11.2 ^a^
AS-DP-U	29.1 ^a^	6.2 ^a^	9.0 ^b^	4.3 ^a^	3.4 ^b^

The data are presented as the mean ± SE. Different letters in the same column represent significant differences according to Tukey’s pairwise a posteriori test after ANOVA, considering *p* < 0.05.

**Table 3:** Mass characterization of PP-U and fractions depolymerized by H_2_O_2_ from EAE by high performance size exclusion chromatography (HPSEC).

	M_n_ (kDa)	M_w_ (kDa)	I
**PP-U**	1651	2290	1.4
**DEP-H1 PP-U**	1480	2154	1.5
**DEP-H3 PP-U**	1343	1943	1.5
**DEP-H7 PP-U**	128	510	4.0
**DEP-HD PP-U**	7.0	8.7	1.2

M_n_: number-averaged molecular weight; M_w_: weight-averaged molecular weight; I: polydispersity index.

**Table 4:** Biochemical composition (% dw) of low-molecular-weight polysaccharides (LMWPs) from EAE.

	Carbohydrates	Uronic Acids	Sulfate Groups	Proteins
**DEP-AD PP-U**	30.4 ± 0.2 ^a^	30.8 ± 1.0 ^a^	nd	12.8 ± 1.0 ^a^
**DEP-HD PP-U**	24.4 ± 0.4 ^b^	21.6 ± 0.4 ^b^	6.6 ± 0.1	16.8 ± 0.4 ^a^

nd: not detected. Values are the mean ± SE (standard error). Different letters in the same column represent significant differences according to Tukey’s pairwise a posteriori test after ANOVA, considering *p* < 0.05.

**Table 5:** Monosaccharide composition (% carbohydrates) of LMWPs from EAE.

	Rhamnose	Galactose	Glucose	Xylose	Glucuronic Acid
**DEP-AD PP-U**	55.4 ± 2.3 ^a^	1.7 ± 0.1 ^a^	7.0 ± 0.4 ^a^	2.6 ± 0.2 ^a^	11.0 ± 0.7 ^a^
**DEP-HD PP-U**	44.9 ^a^	1.0 ^b^	4.9 ^a^	2.1 ^a^	7.5 ^b^

The data are presented as the mean ± SE. Different letters in the same column represent significant differences according to Tukey’s pairwise a posteriori test after ANOVA, considering *p* < 0.05.

**Table 6:** Conditions of depolymerization by H_2_O_2_ of polysaccharides from *Ulva* sp.

Fractions	Temperature (°C)	Time (h)	Dialysis (500–1000 Da)
**DEP-H1 PP-U**	35	1	No
**DEP-H3 PP-U**	Step 1: 35 Step 2: 50	1 2	No
**DEP-H7 PP-U**	Step 1: 35 Step 2: 50	1 6	No
**DEP-HD PP-U**	50	24	Yes

**Table 7:** Chromatographic columns used for HPSEC analysis.

	Chromatographic Columns
**PP-U**	G6000PW_XL_ G4000PW_XL_ G3000PW_XL_
**DEP-H1 PP-U**	
**DEP-H3 PP-U**	
**DEP-H7 PP-U**	
**DEP-HD PP-U**	G3000PW_XL_
**DEP-AD PP-U**	

## Supplementary Materials

The following are available online at https://www.mdpi.com/2218-1989/9/9/182/s1. Figure S1: Effect of *Ulva* sp. extracts and fractions on lipoxygenase inhibition; Figure S2: Effect of polysaccharidic extracts and fractions from *Ulva* sp. from maceration on fibroblast proliferation evaluated by the WST-1 assay after incubating cells in the presence of extracts (50–1000 μg/mL) for 24 and 48 h; Figure S3: Effect of depolymerized fractions from *Ulva* sp. from maceration on fibroblast proliferation evaluated by WST-1 assay after incubating cells in the presence of extracts (50–1000 μg/mL) for 24 and 48 h; Table S1a: Biochemical composition (% dw) of crude ulvans and high-molecular-weight polysaccharides (HMWPs); Table S1b: Biochemical composition (% dw) of low-molecular-weight polysaccharides (LMWPs); Table S2a: Monosaccharide composition (%carbohydrates) of crude ulvans and HMWPs; Table S2b: Monosaccharide composition (%carbohydrates) of LMWPs; Table S3: Mass characterization of PP-U and fractions depolymerized by H_2_O_2_ from maceration by HPSEC.
