# Supplementary figures and images for: Production of Active Poly- and Oligosaccharidic Fractions from Ulva sp. by Combining Enzyme-Assisted Extraction (EAE) and Depolymerization
Source: Metabolites. 2019 Sep 12;9(9):182. doi: 10.3390/metabo9090182 (PMC6780239; doi:10.3390/metabo9090182)

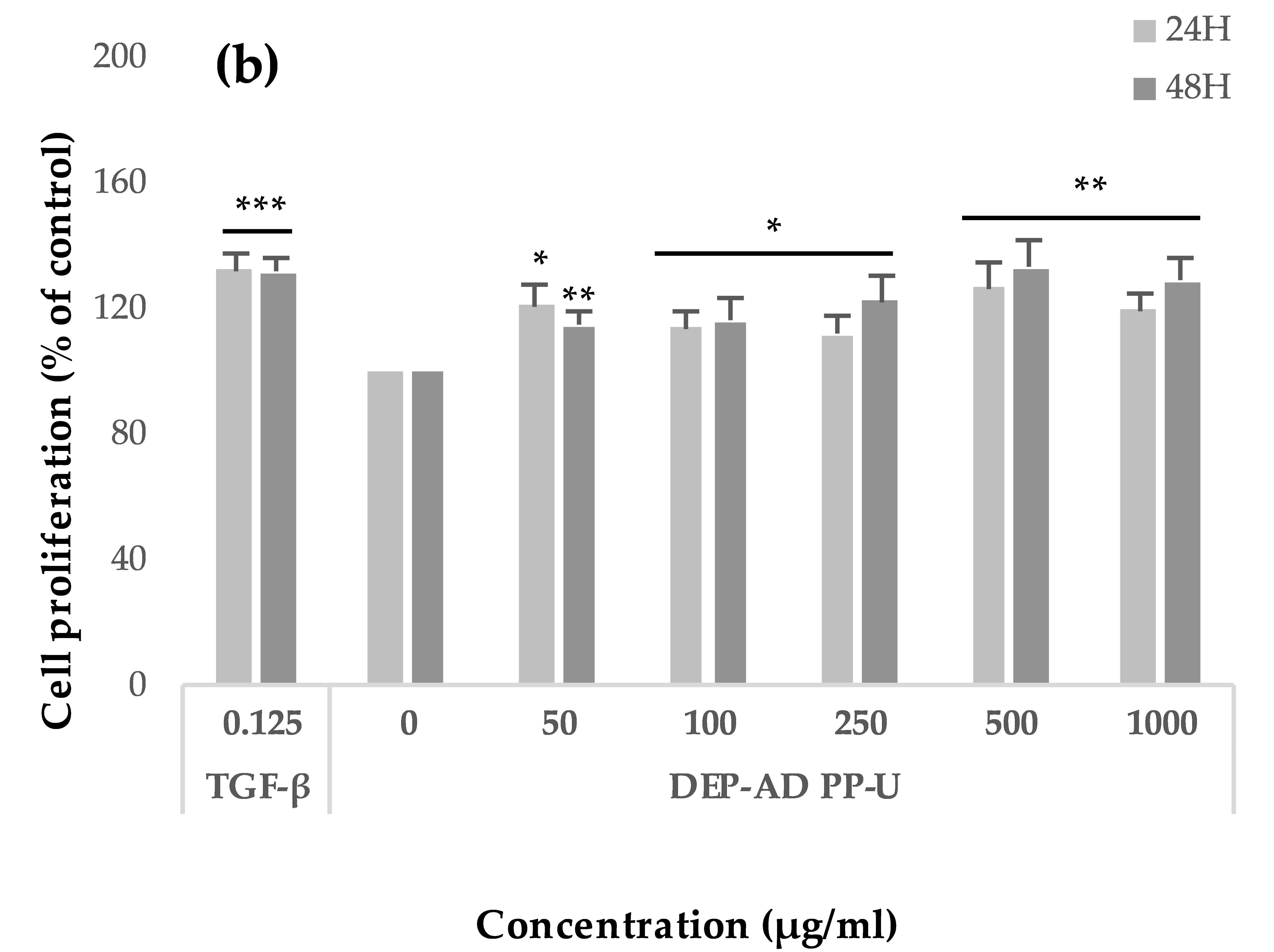

Supplement: Supplementary file 1 [file metabolites-09-00182-s001.zip › Figures and Tables TIF/Figure 5b.tif]

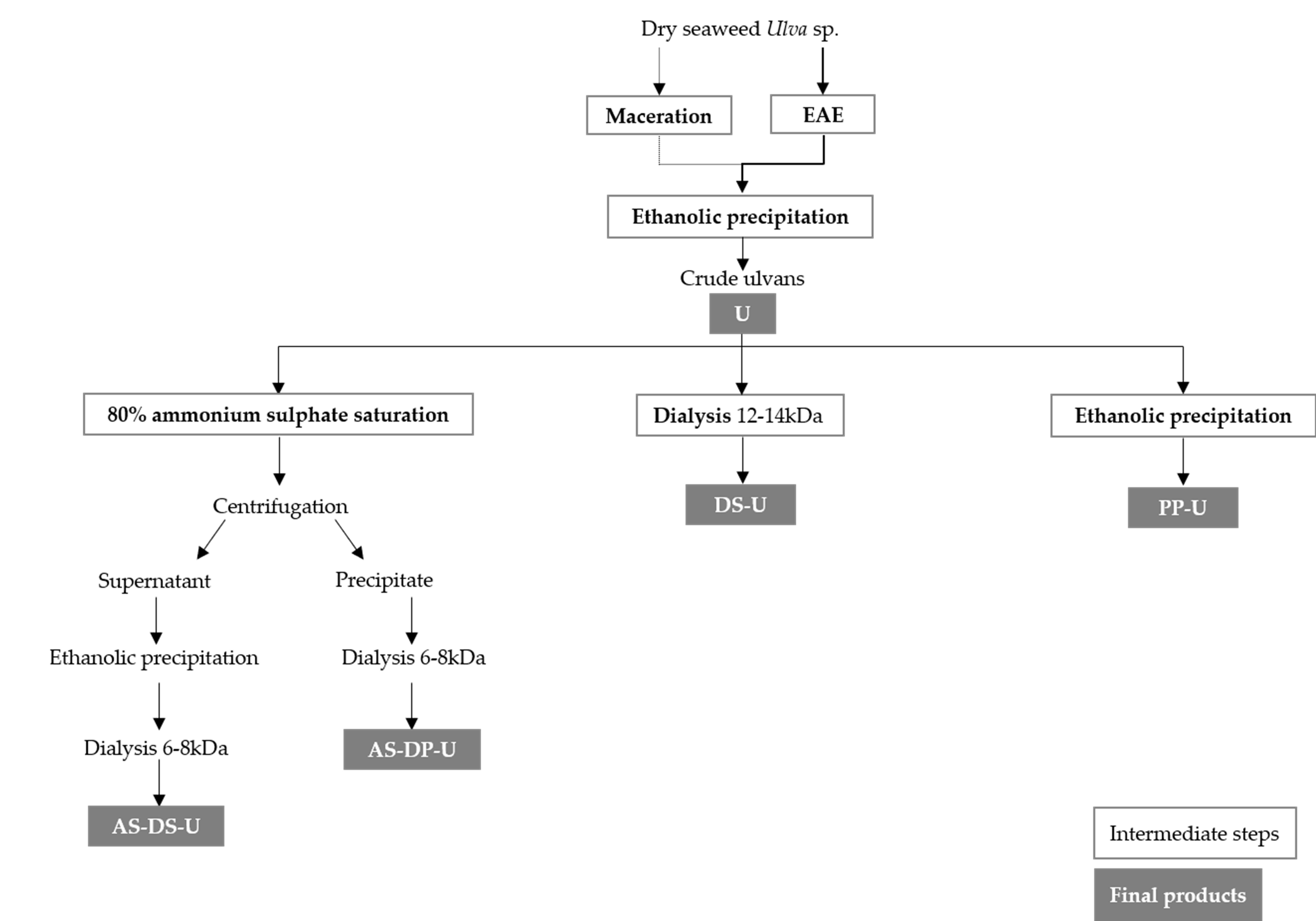

Supplement: Supplementary file 1 [file metabolites-09-00182-s001.zip › Figures and Tables TIF/Figure 6.tif]

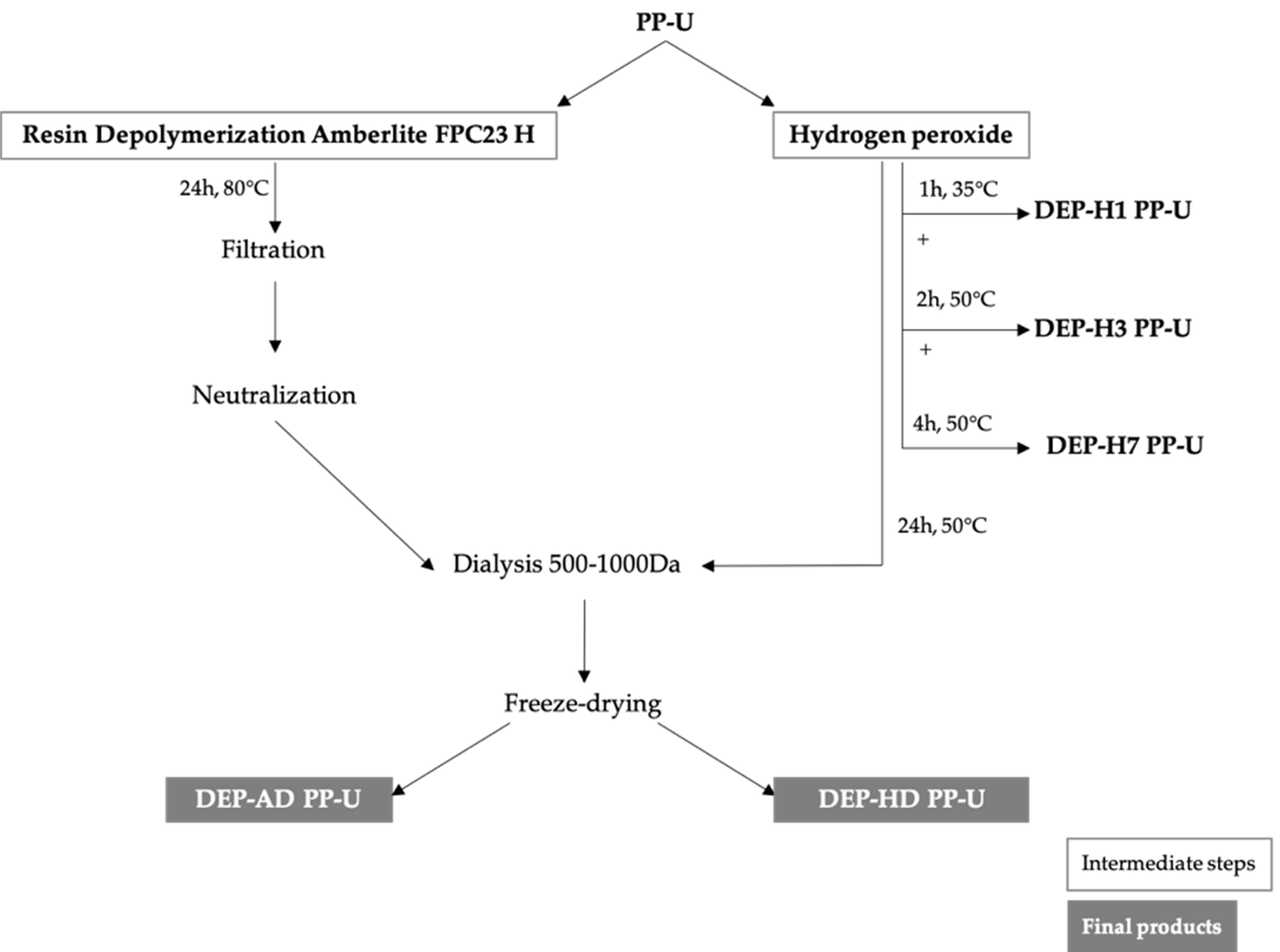

Supplement: Supplementary file 1 [file metabolites-09-00182-s001.zip › Figures and Tables TIF/Figure 7.tif]

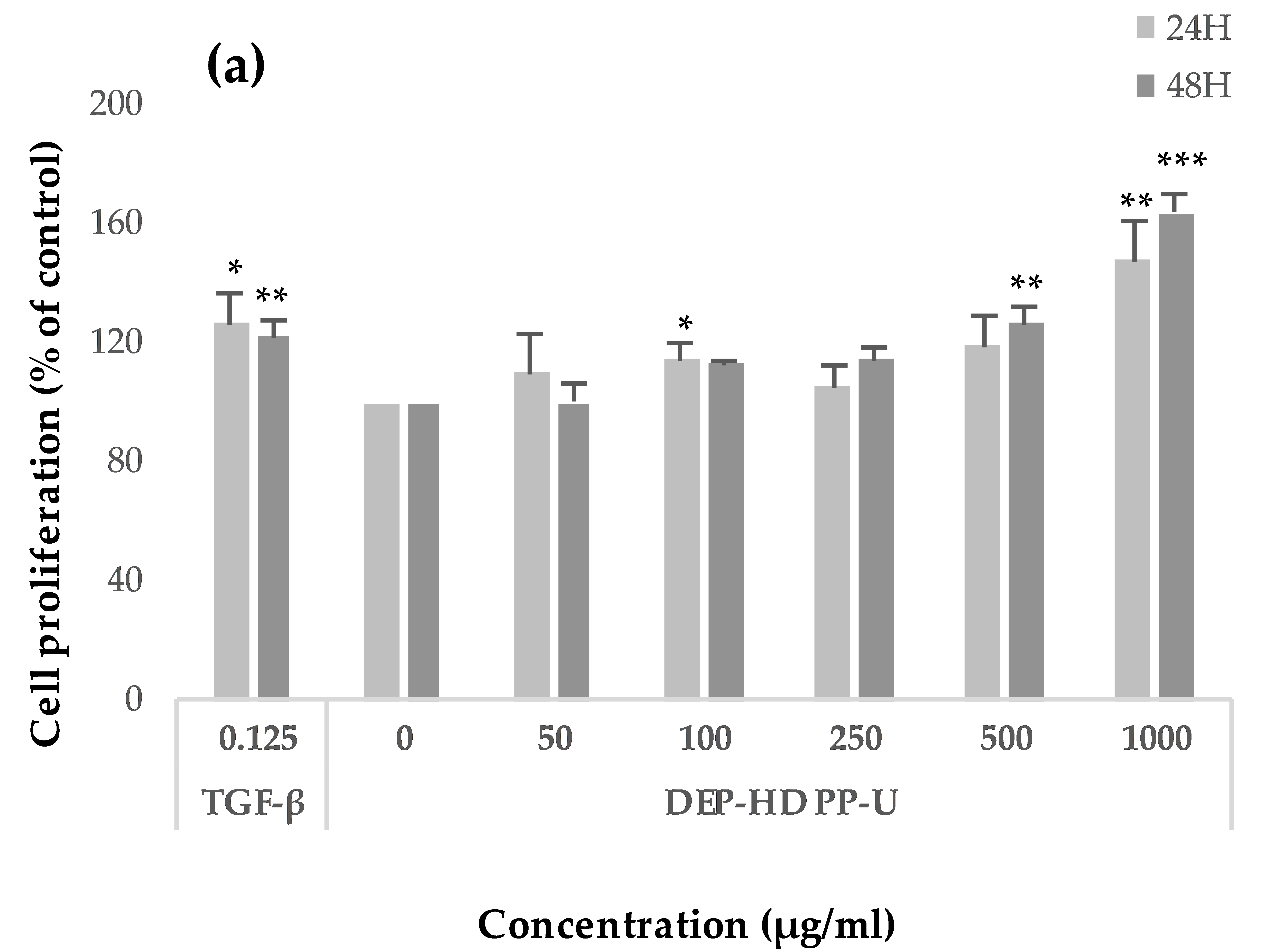

Supplement: Supplementary file 1 [file metabolites-09-00182-s001.zip › Figures and Tables TIF/Figure 5a.tif]

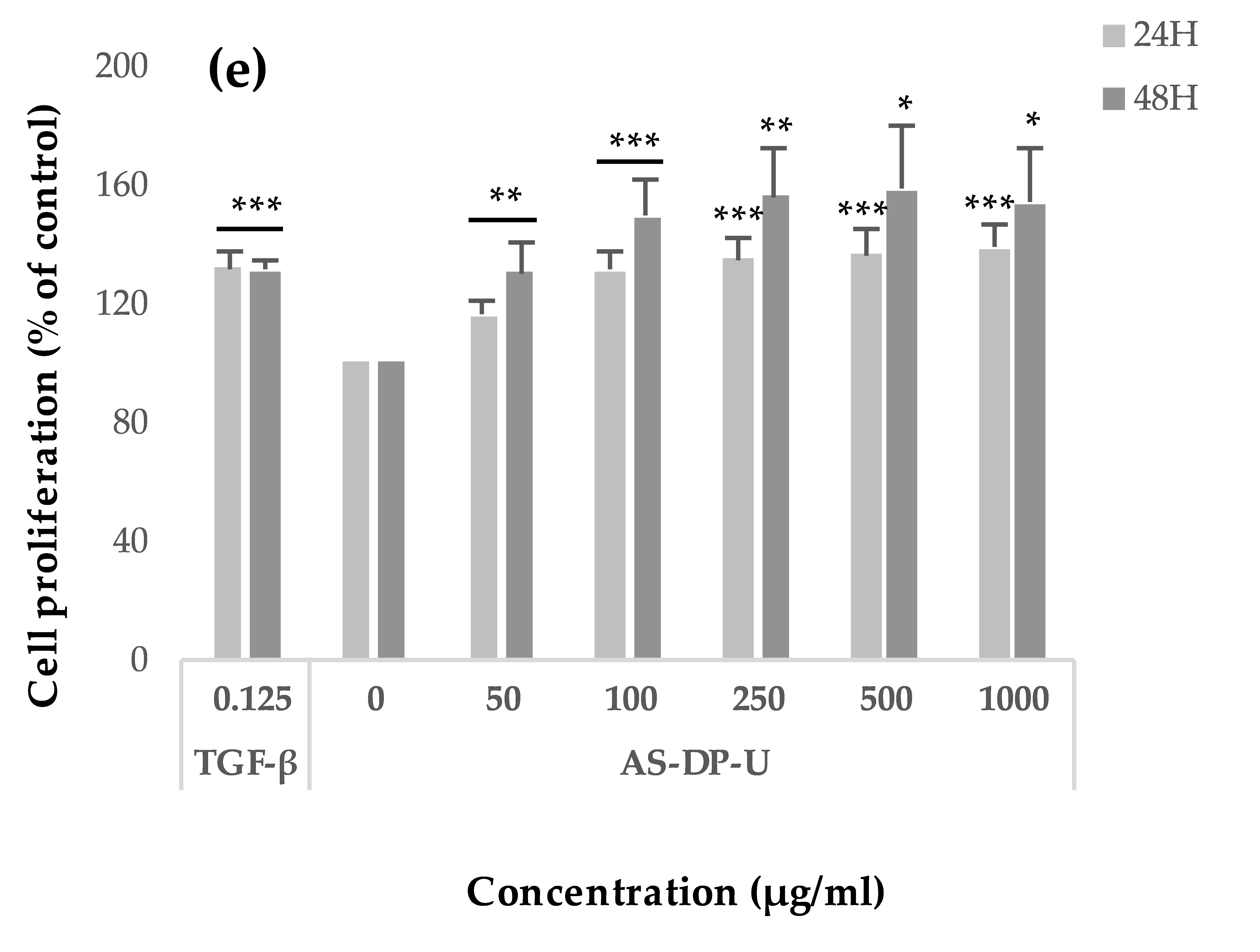

Supplement: Supplementary file 1 [file metabolites-09-00182-s001.zip › Figures and Tables TIF/Figure 4e.tif]

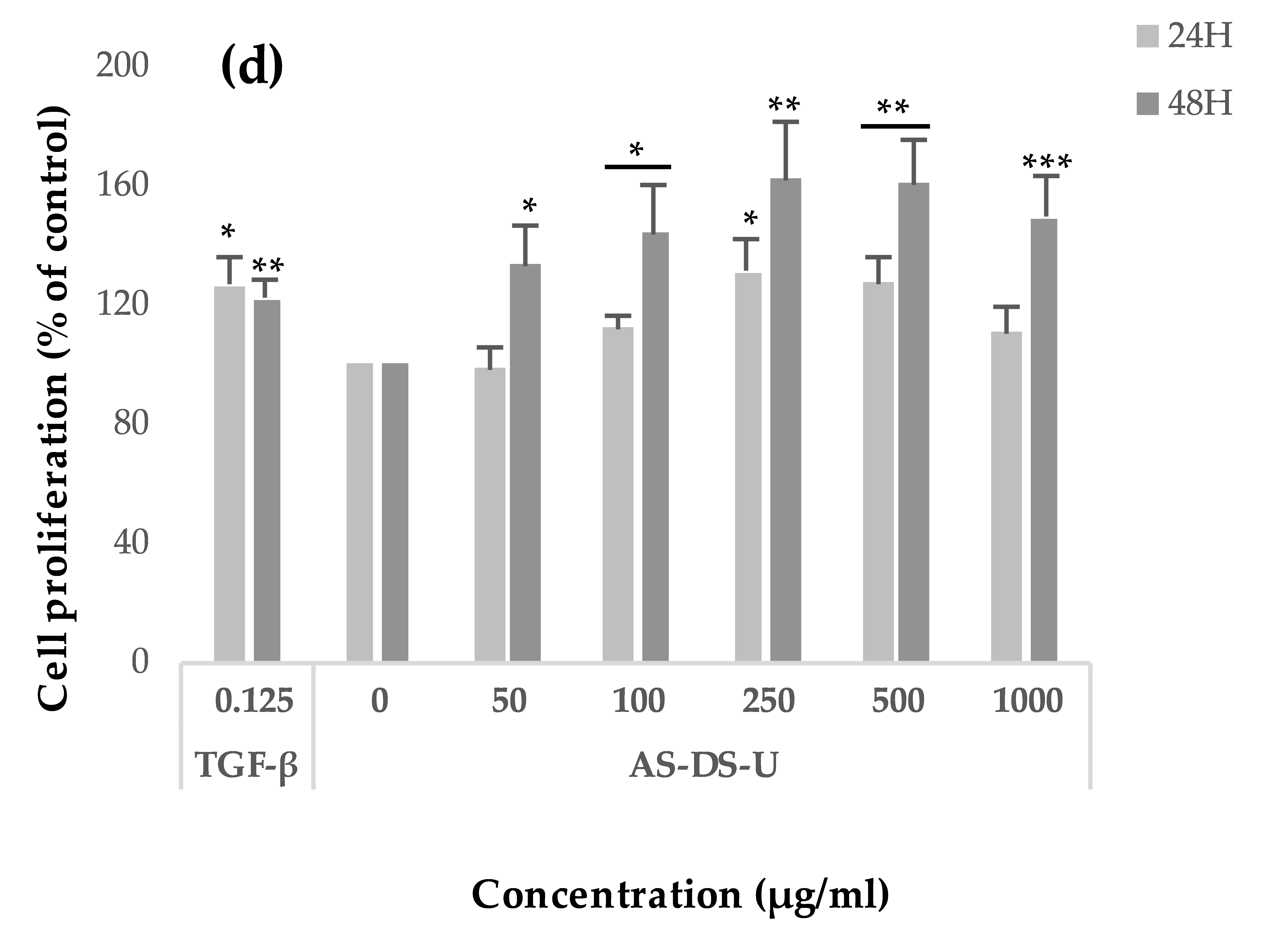

Supplement: Supplementary file 1 [file metabolites-09-00182-s001.zip › Figures and Tables TIF/Figure 4d.tif]

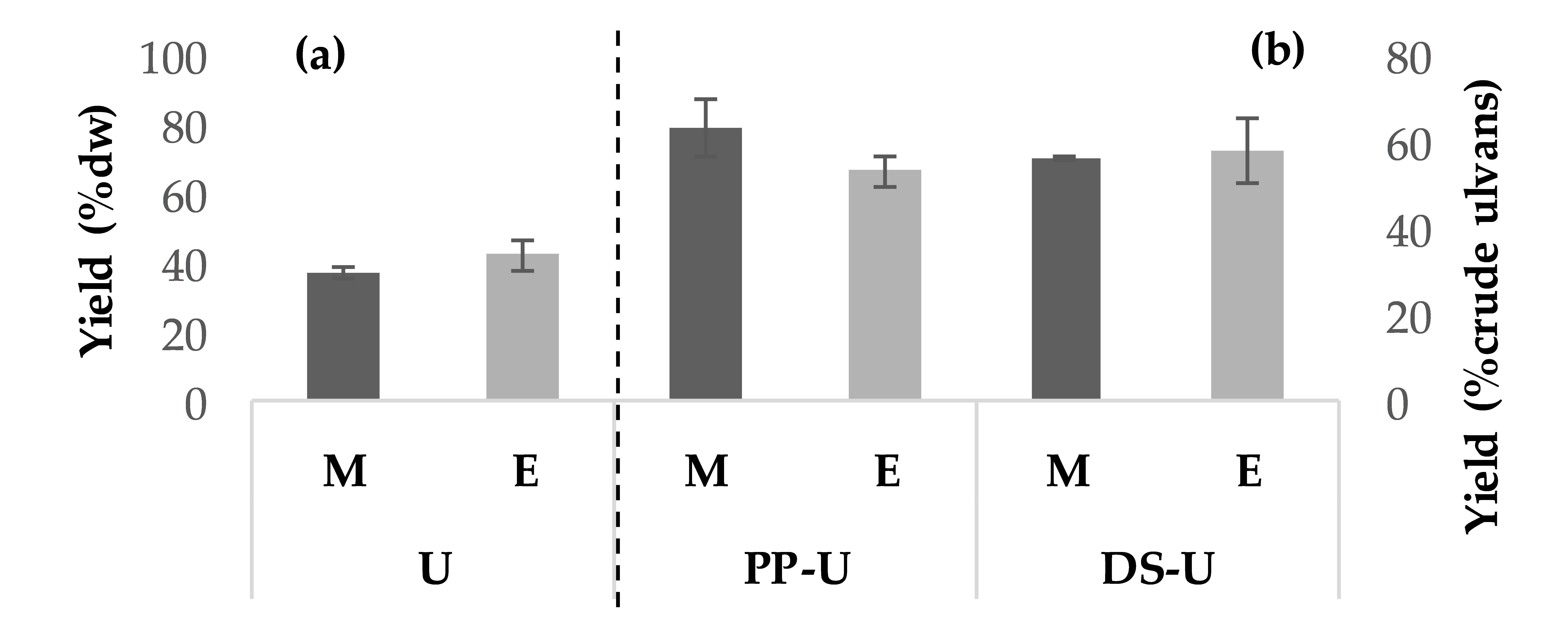

Supplement: Supplementary file 1 [file metabolites-09-00182-s001.zip › Figures and Tables TIF/Figure 1.tif]

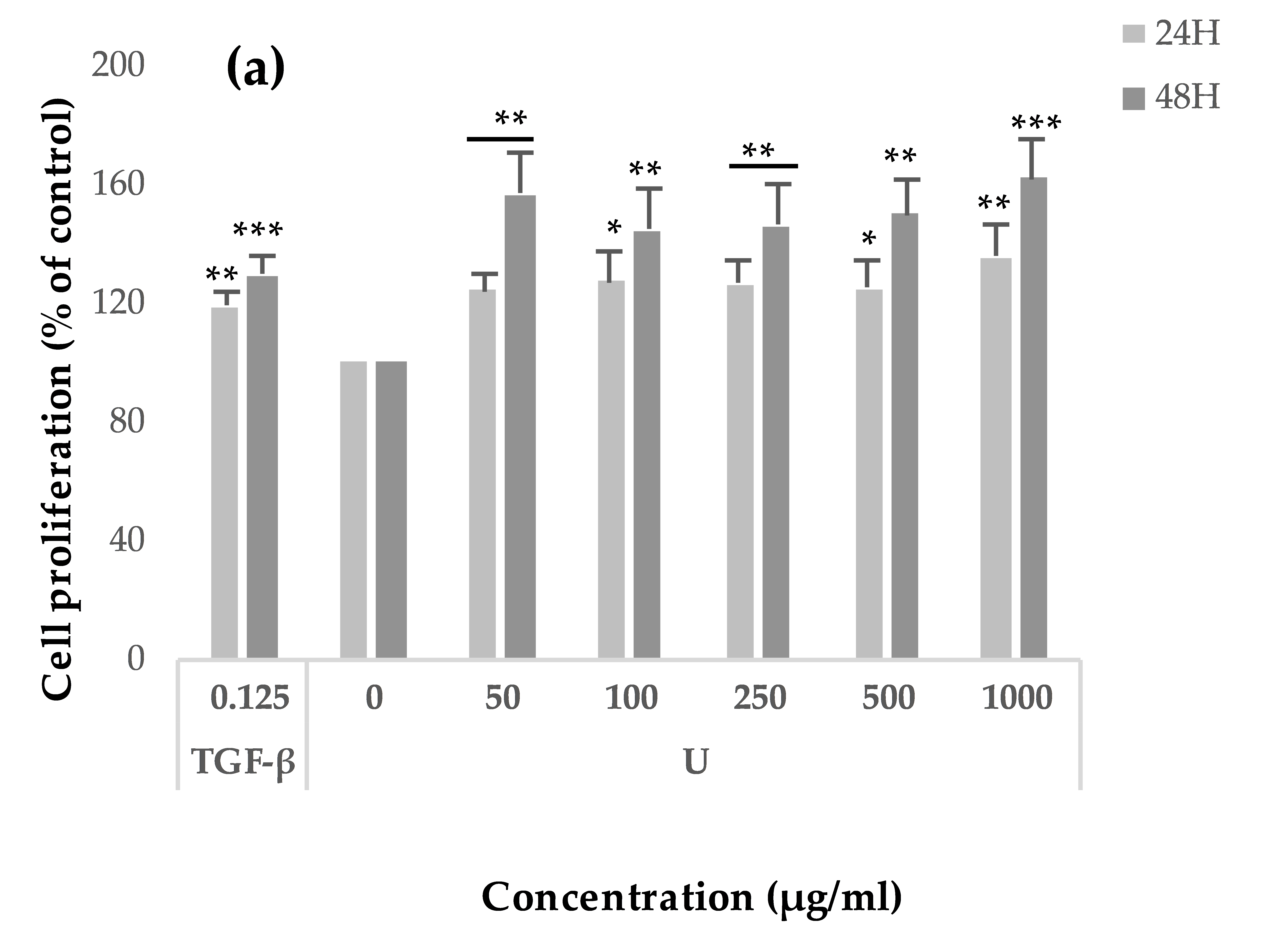

Supplement: Supplementary file 1 [file metabolites-09-00182-s001.zip › Figures and Tables TIF/Figure 4a.tif]

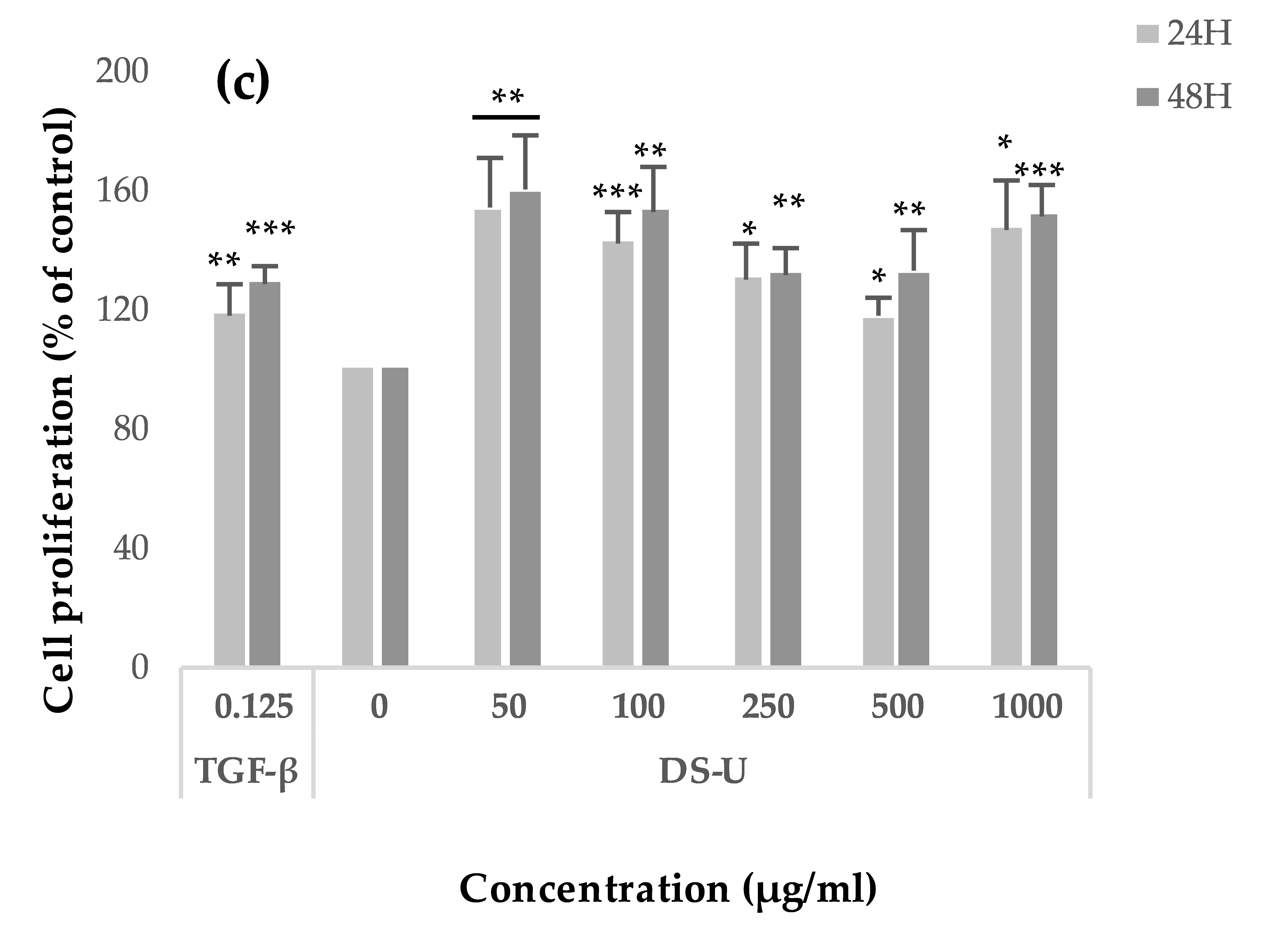

Supplement: Supplementary file 1 [file metabolites-09-00182-s001.zip › Figures and Tables TIF/Figure 4c.tif]

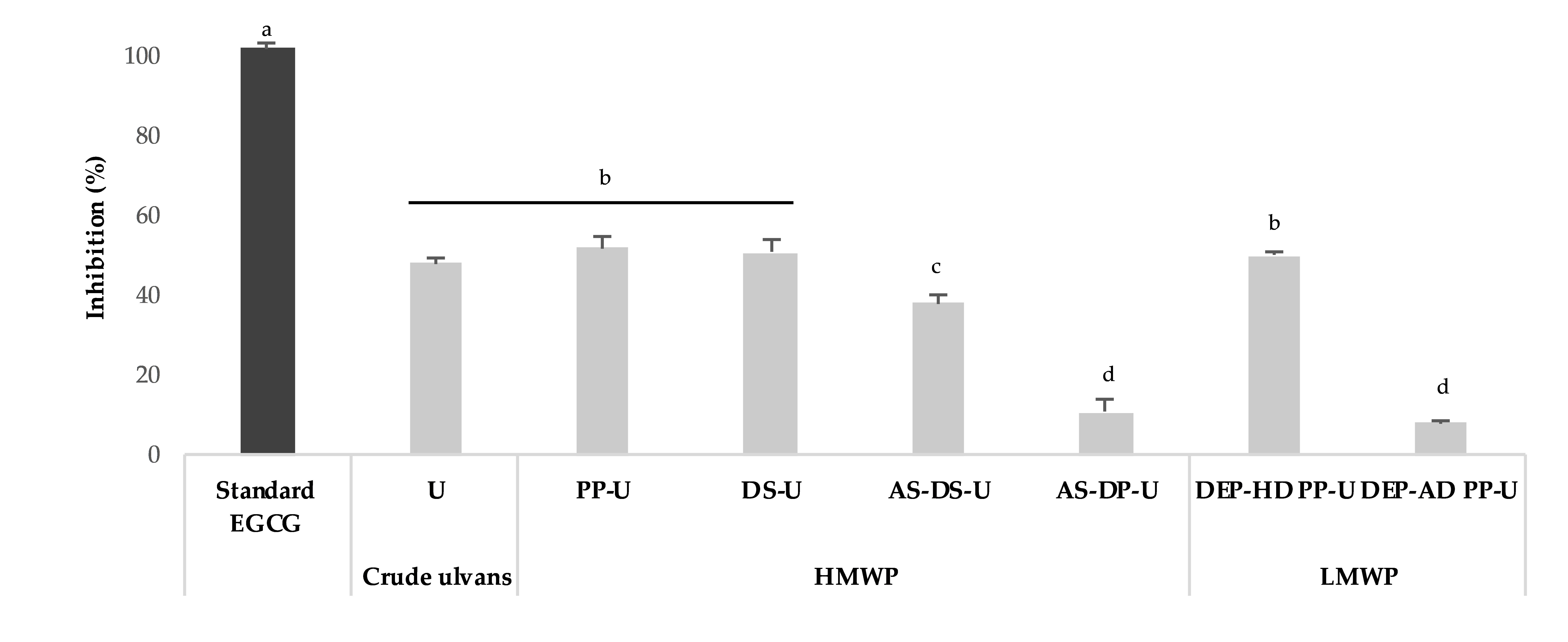

Supplement: Supplementary file 1 [file metabolites-09-00182-s001.zip › Figures and Tables TIF/Figure 3.tif]

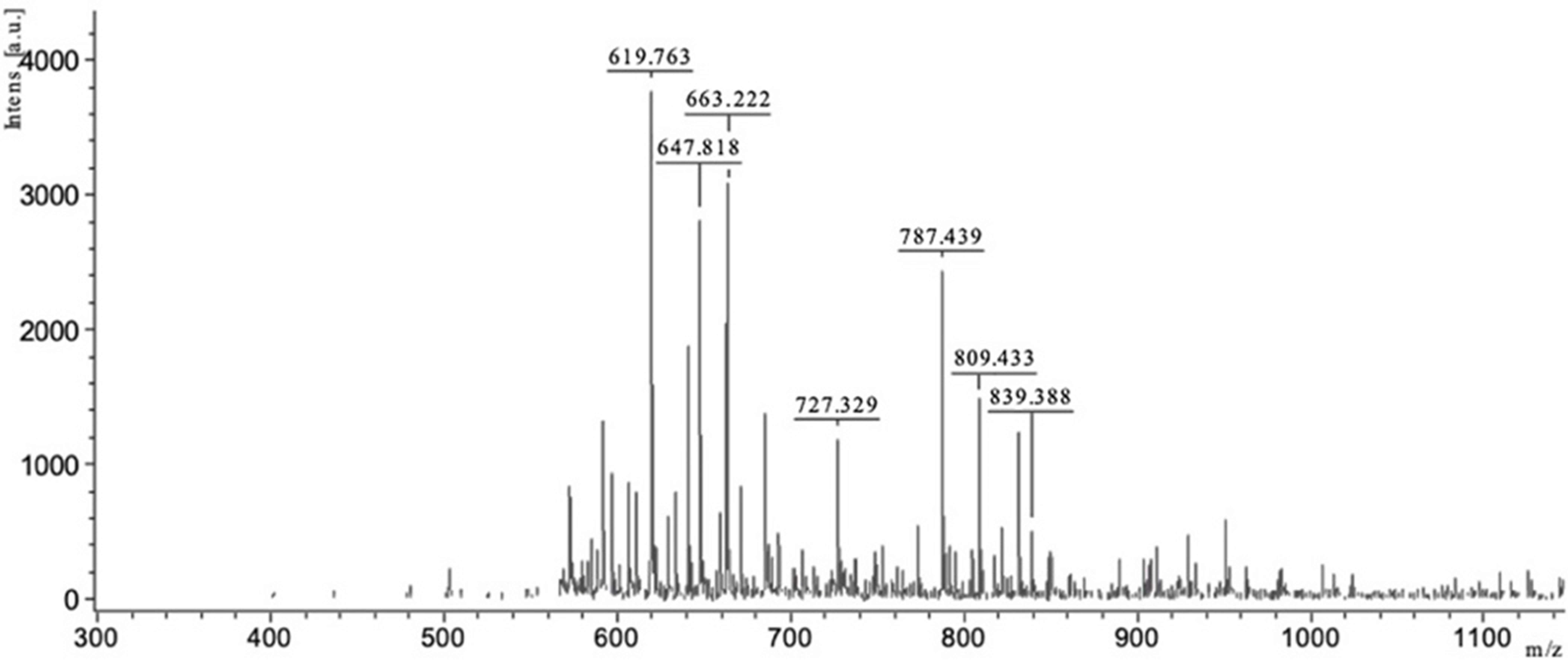

Supplement: Supplementary file 1 [file metabolites-09-00182-s001.zip › Figures and Tables TIF/Figure 2.tif]

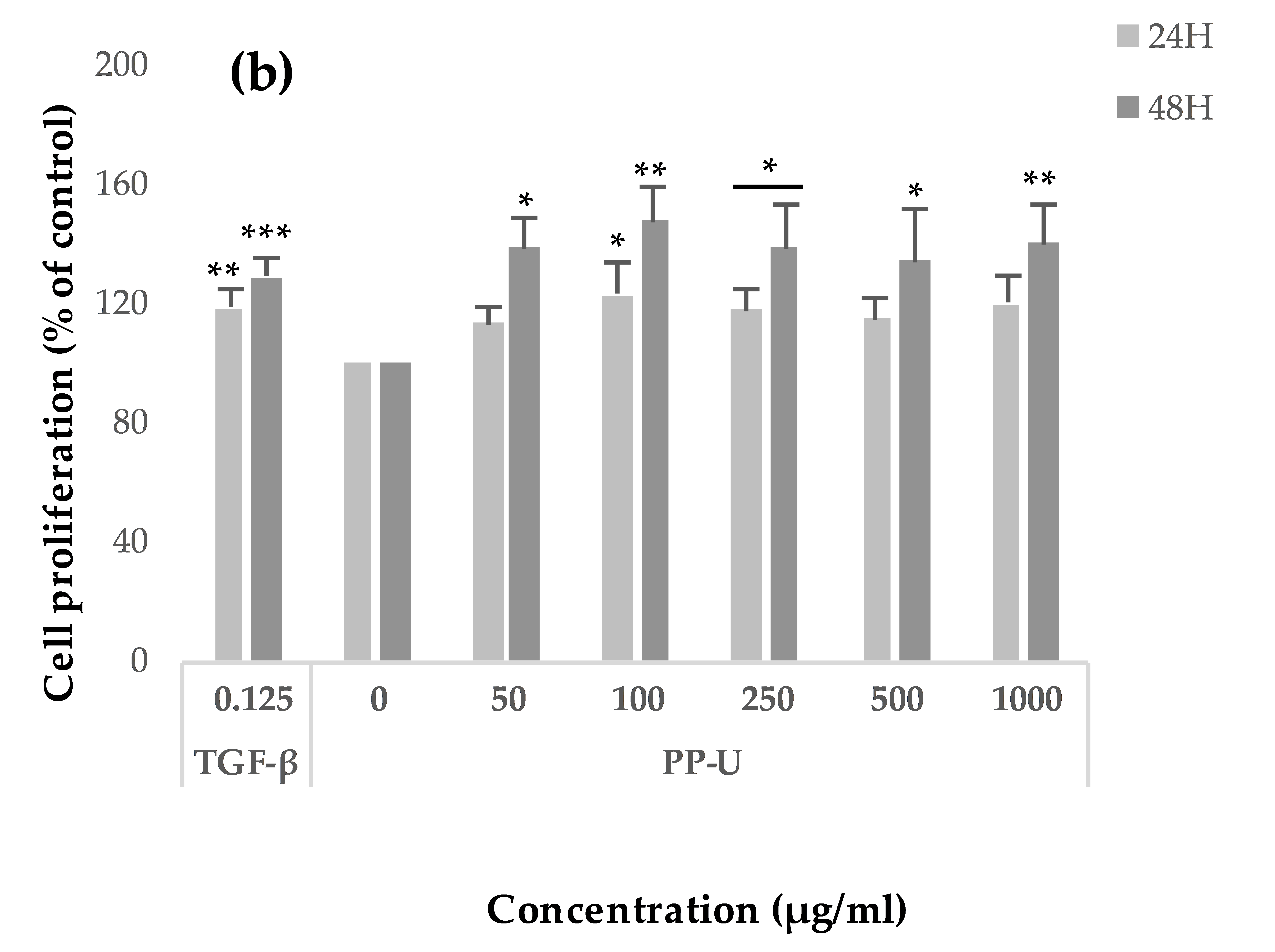

Supplement: Supplementary file 1 [file metabolites-09-00182-s001.zip › Figures and Tables TIF/Figure 4b.tif]

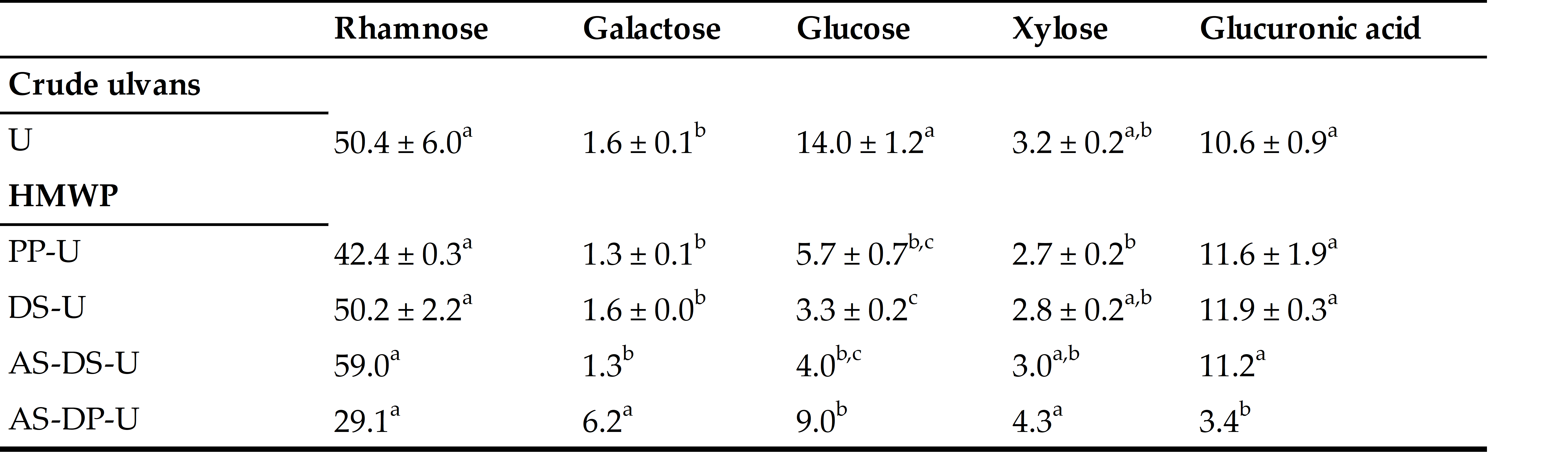

Supplement: Supplementary file 1 [file metabolites-09-00182-s001.zip › Figures and Tables TIF/Table 2.tif]

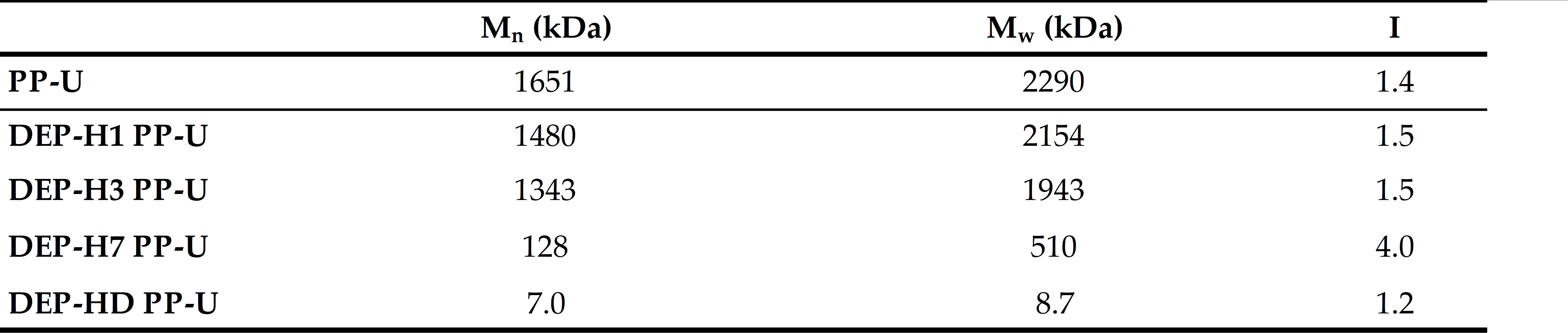

Supplement: Supplementary file 1 [file metabolites-09-00182-s001.zip › Figures and Tables TIF/Table 3.tif]

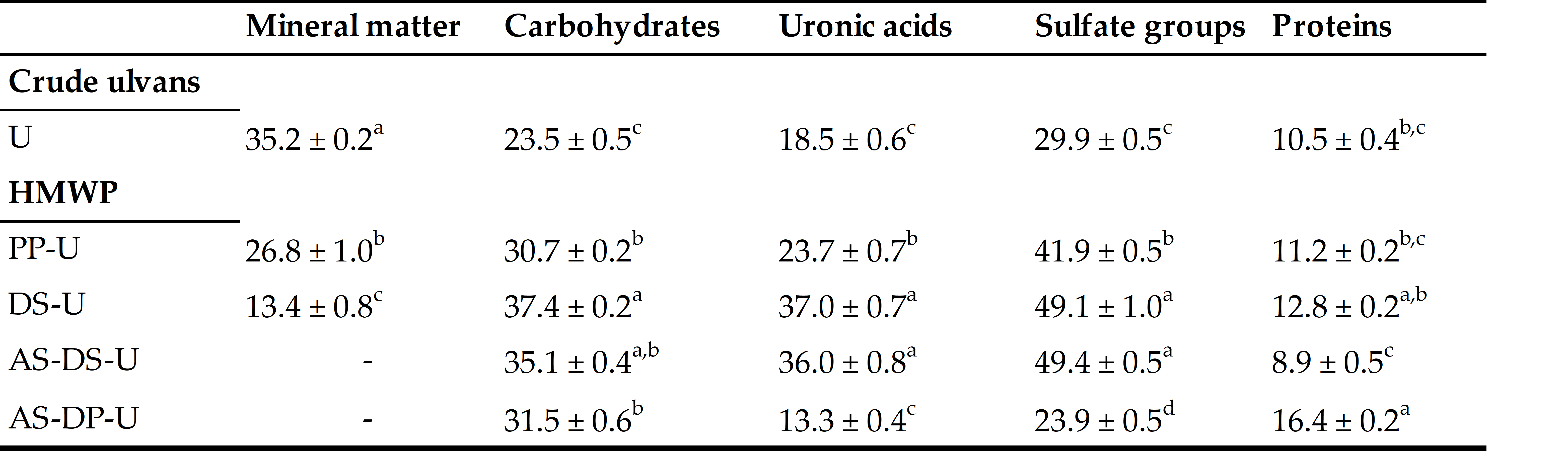

Supplement: Supplementary file 1 [file metabolites-09-00182-s001.zip › Figures and Tables TIF/Table 1.tif]

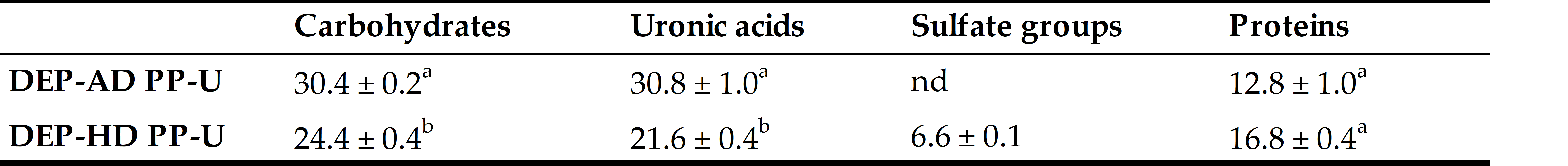

Supplement: Supplementary file 1 [file metabolites-09-00182-s001.zip › Figures and Tables TIF/Table 4.tif]

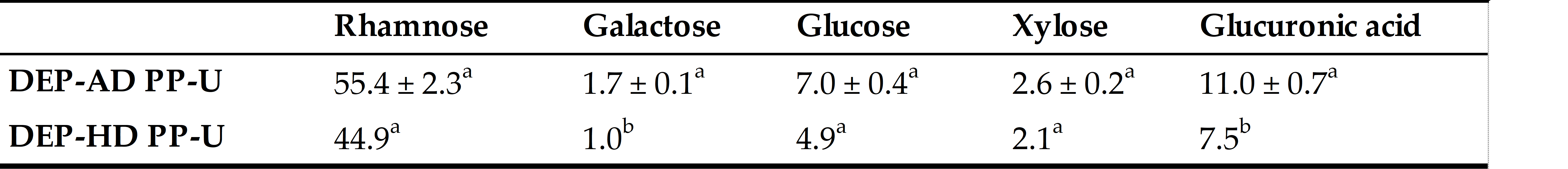

Supplement: Supplementary file 1 [file metabolites-09-00182-s001.zip › Figures and Tables TIF/Table 5.tif]

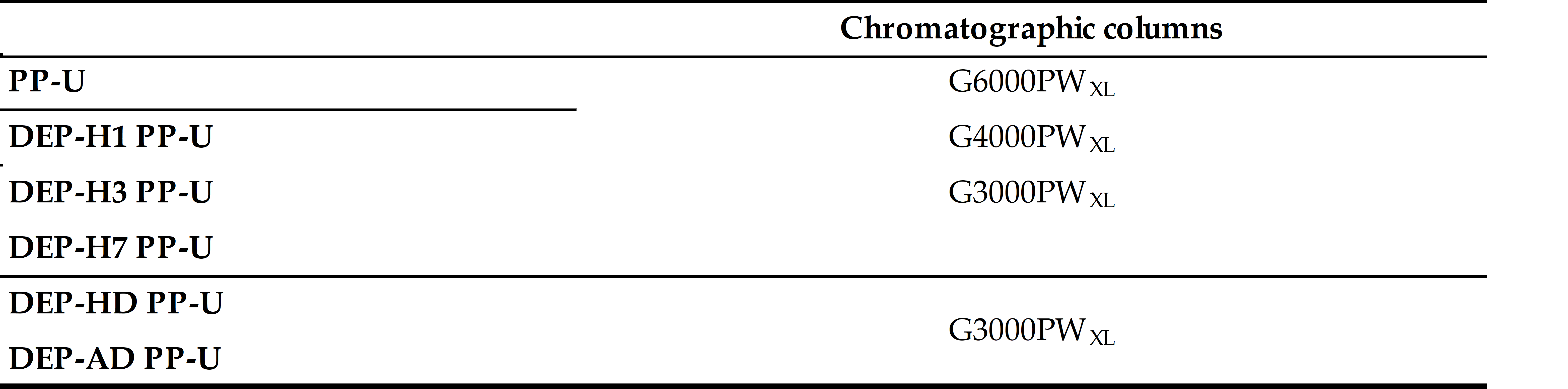

Supplement: Supplementary file 1 [file metabolites-09-00182-s001.zip › Figures and Tables TIF/Table 7.tif]

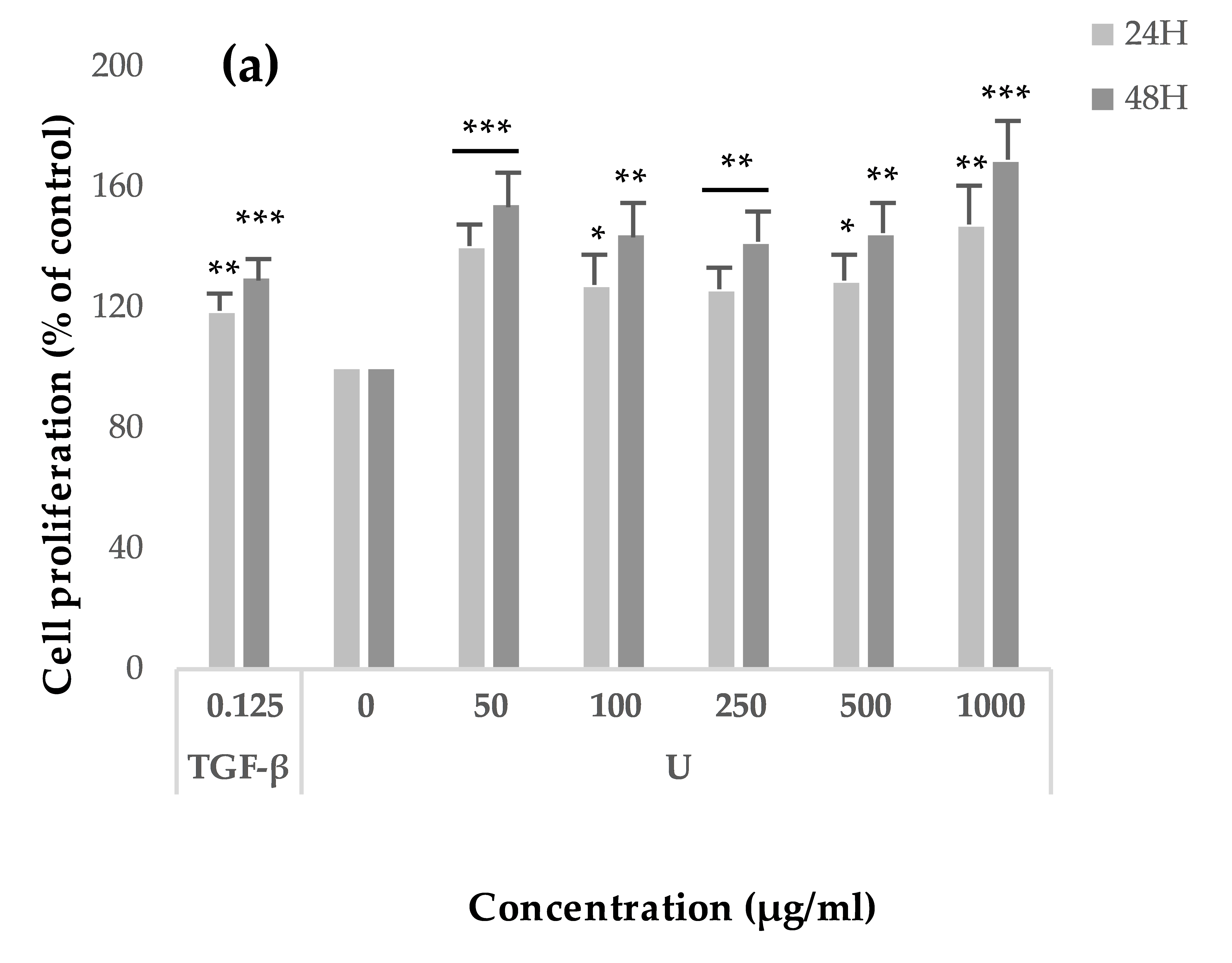

Supplement: Supplementary file 1 [file metabolites-09-00182-s001.zip › Figures and Tables TIF/Supplementary materials/Figure S2a.tif]

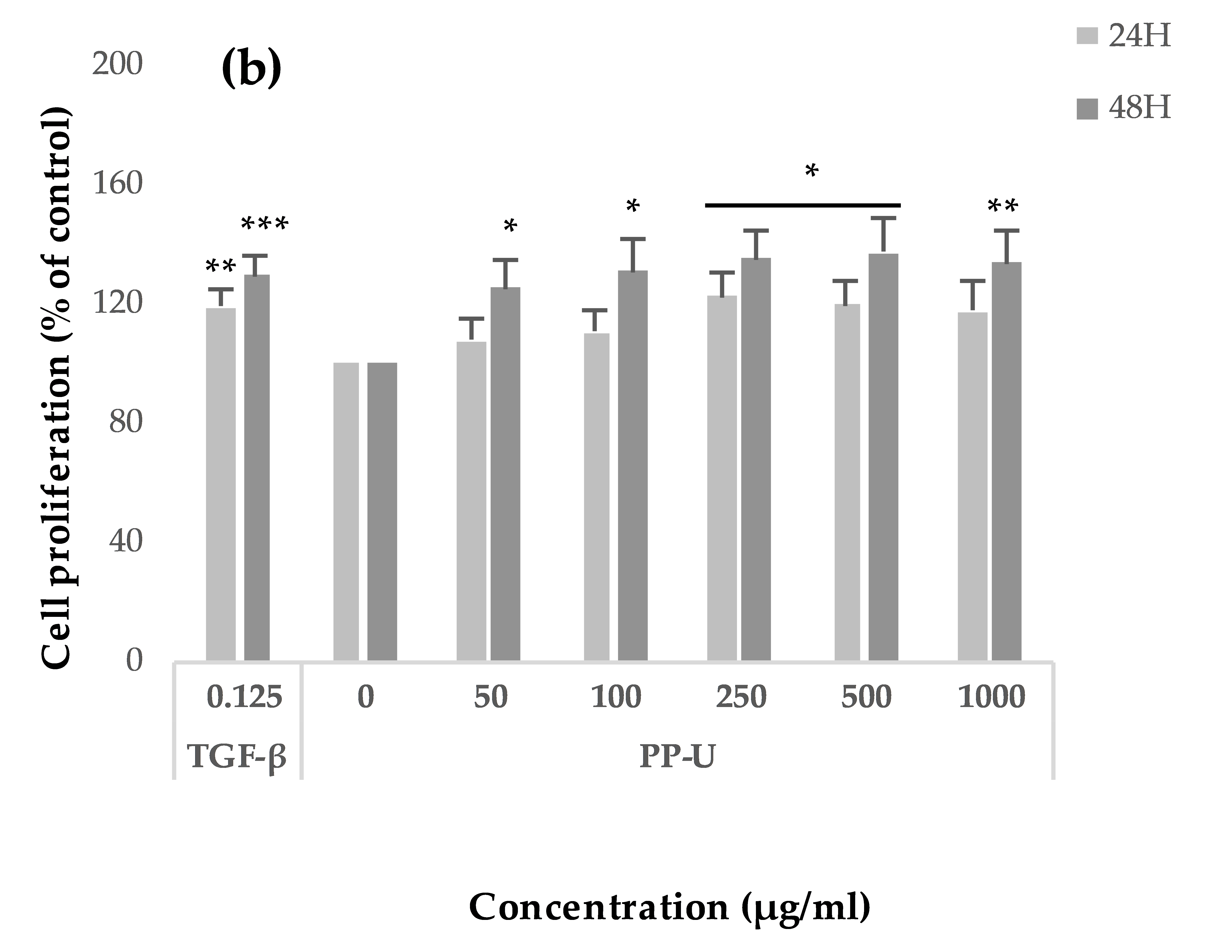

Supplement: Supplementary file 1 [file metabolites-09-00182-s001.zip › Figures and Tables TIF/Supplementary materials/Figure S2b.tif]

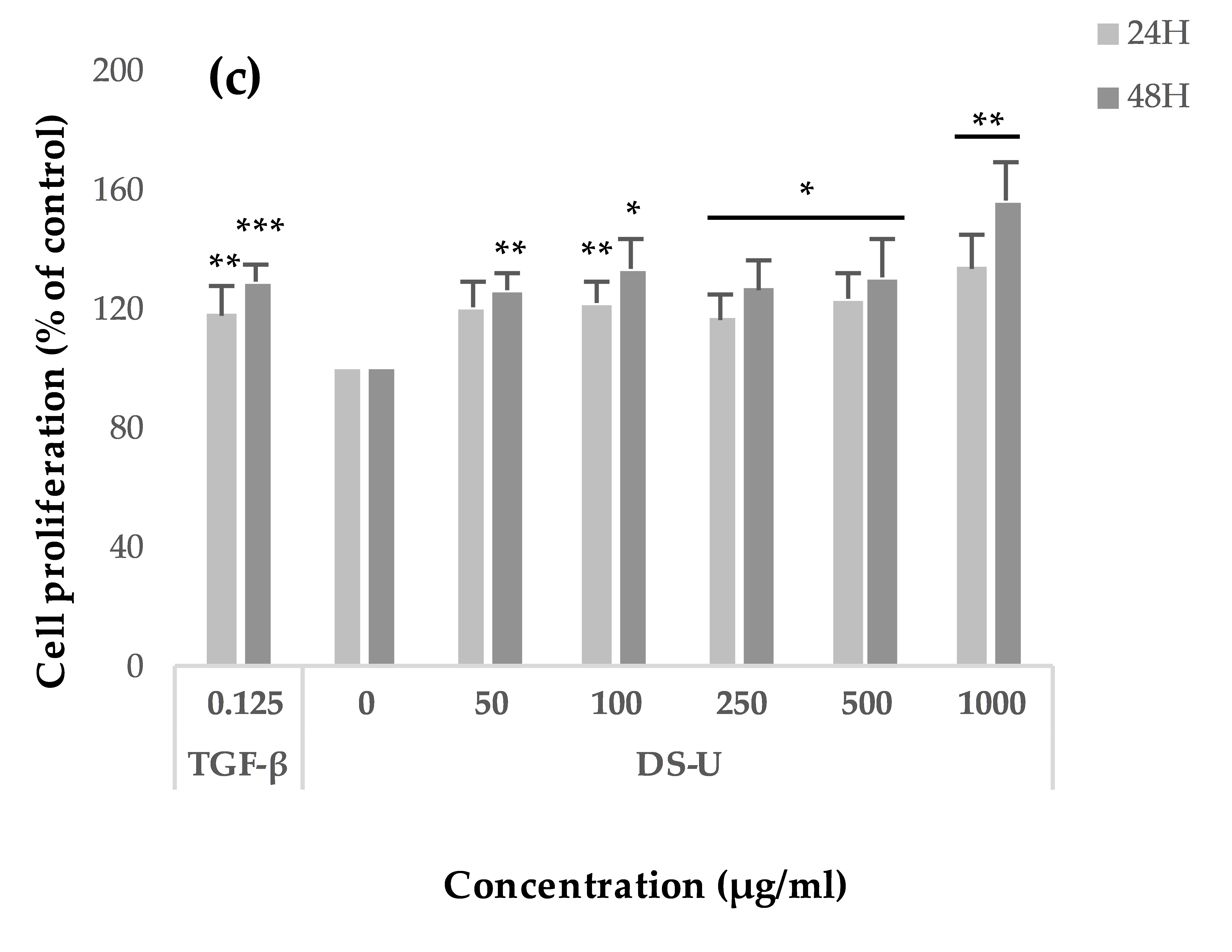

Supplement: Supplementary file 1 [file metabolites-09-00182-s001.zip › Figures and Tables TIF/Supplementary materials/Figure S2c.tif]

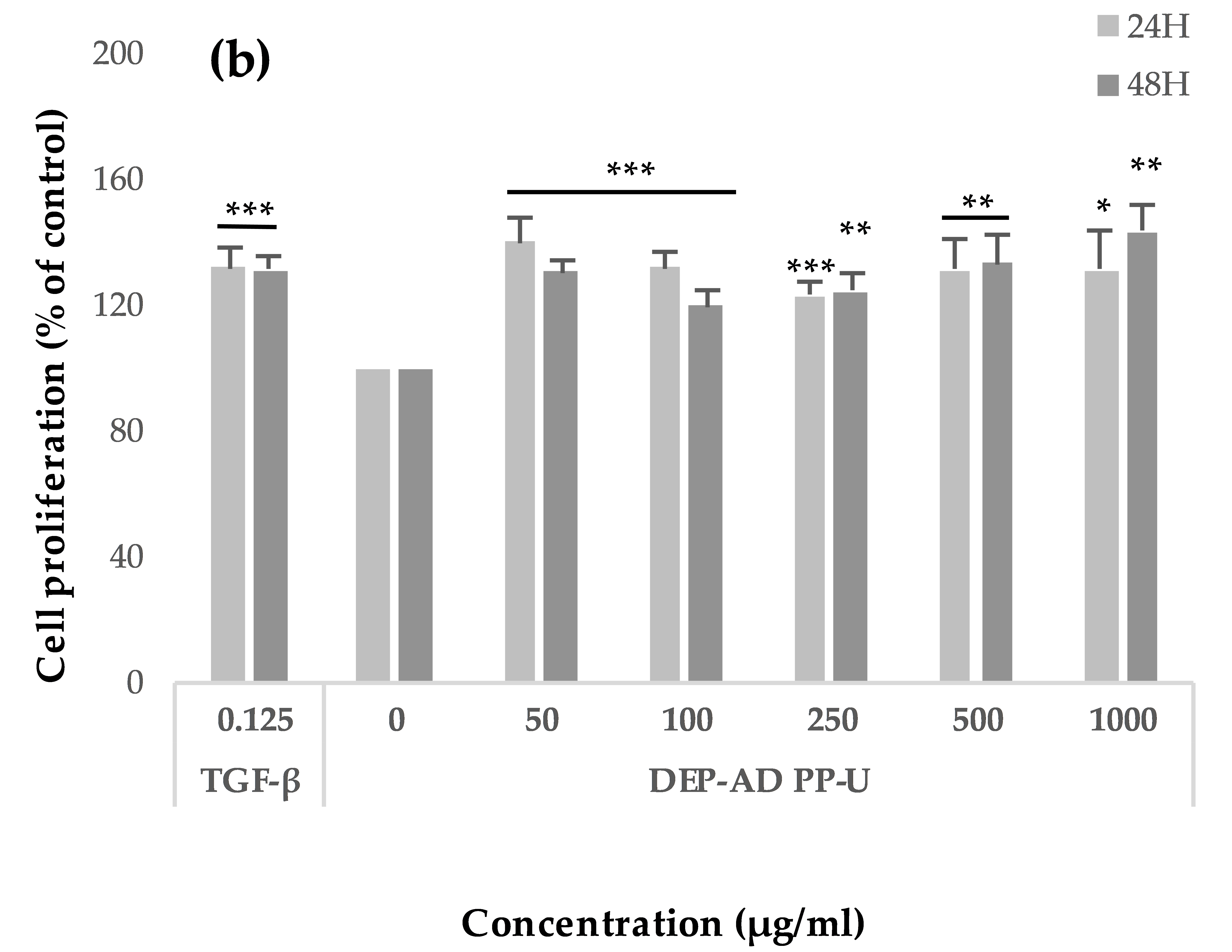

Supplement: Supplementary file 1 [file metabolites-09-00182-s001.zip › Figures and Tables TIF/Supplementary materials/Figure S3b.tif]

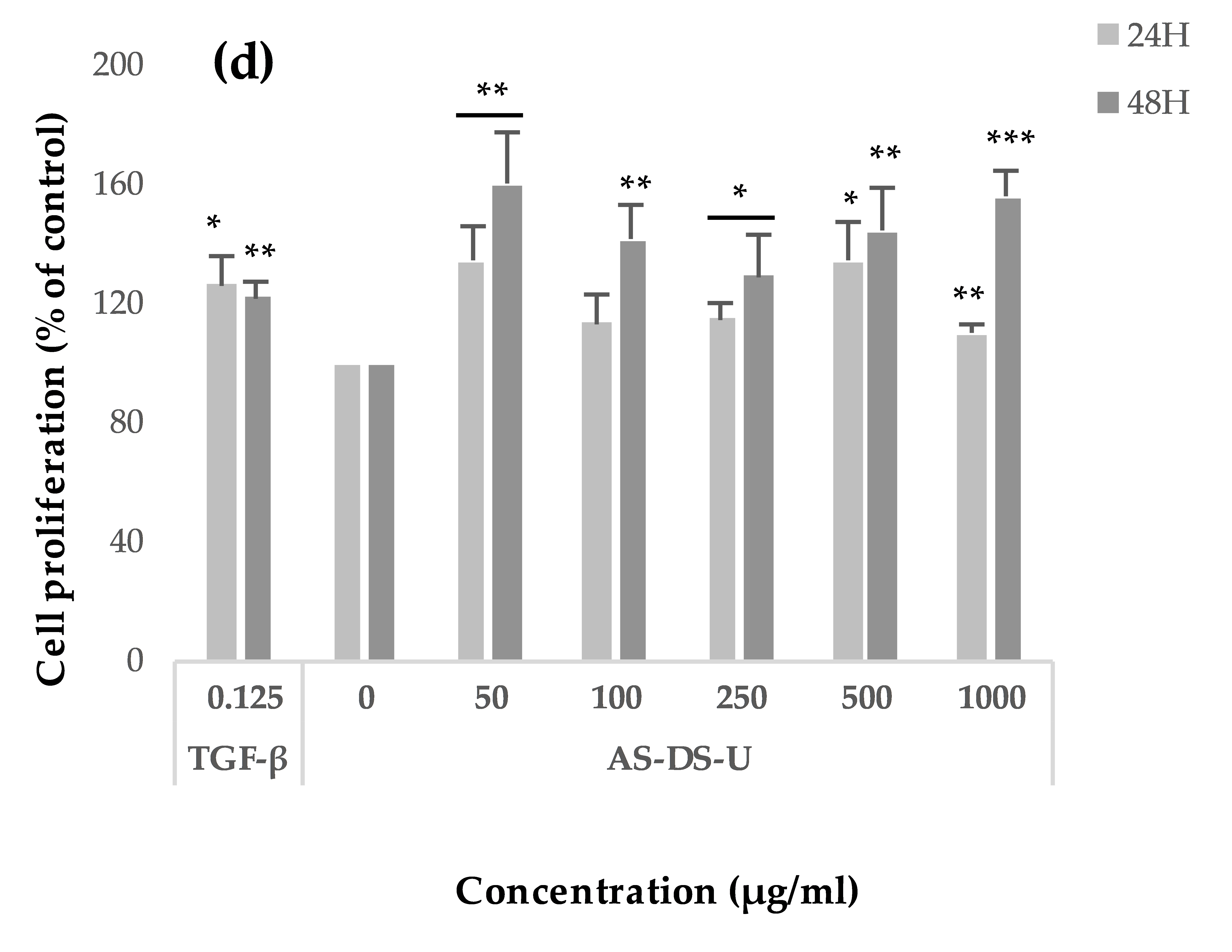

Supplement: Supplementary file 1 [file metabolites-09-00182-s001.zip › Figures and Tables TIF/Supplementary materials/Figure S2d.tif]

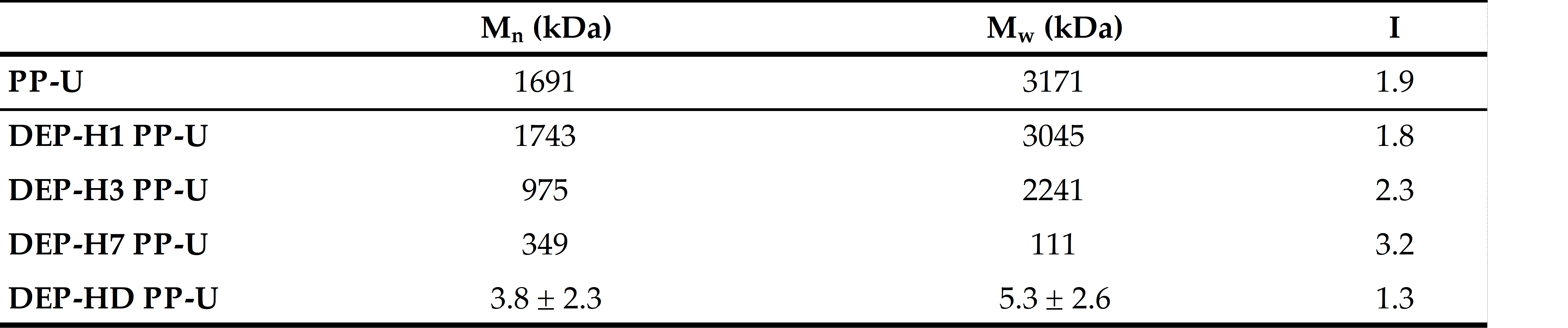

Supplement: Supplementary file 1 [file metabolites-09-00182-s001.zip › Figures and Tables TIF/Supplementary materials/Table S3.tif]

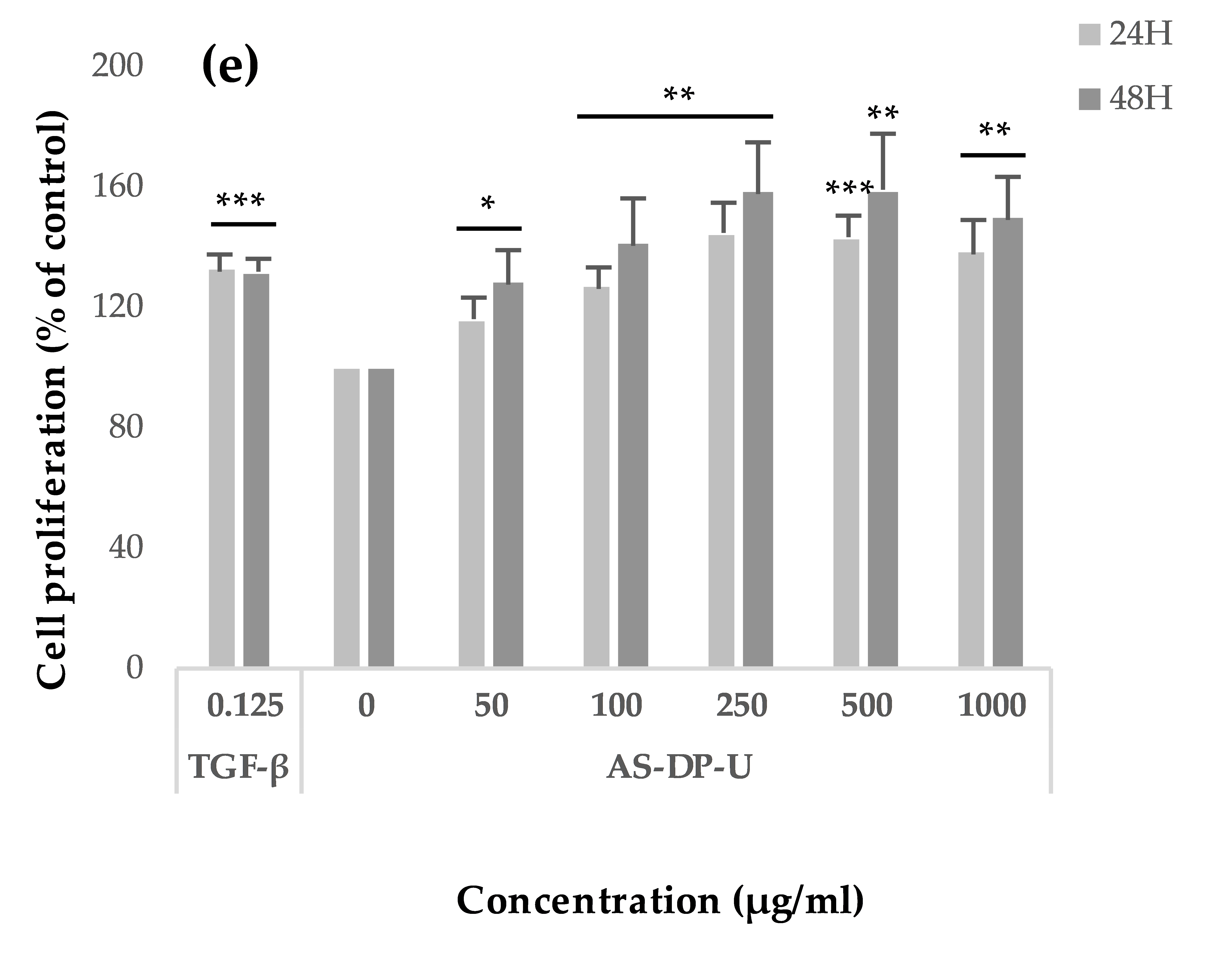

Supplement: Supplementary file 1 [file metabolites-09-00182-s001.zip › Figures and Tables TIF/Supplementary materials/Figure S2e.tif]

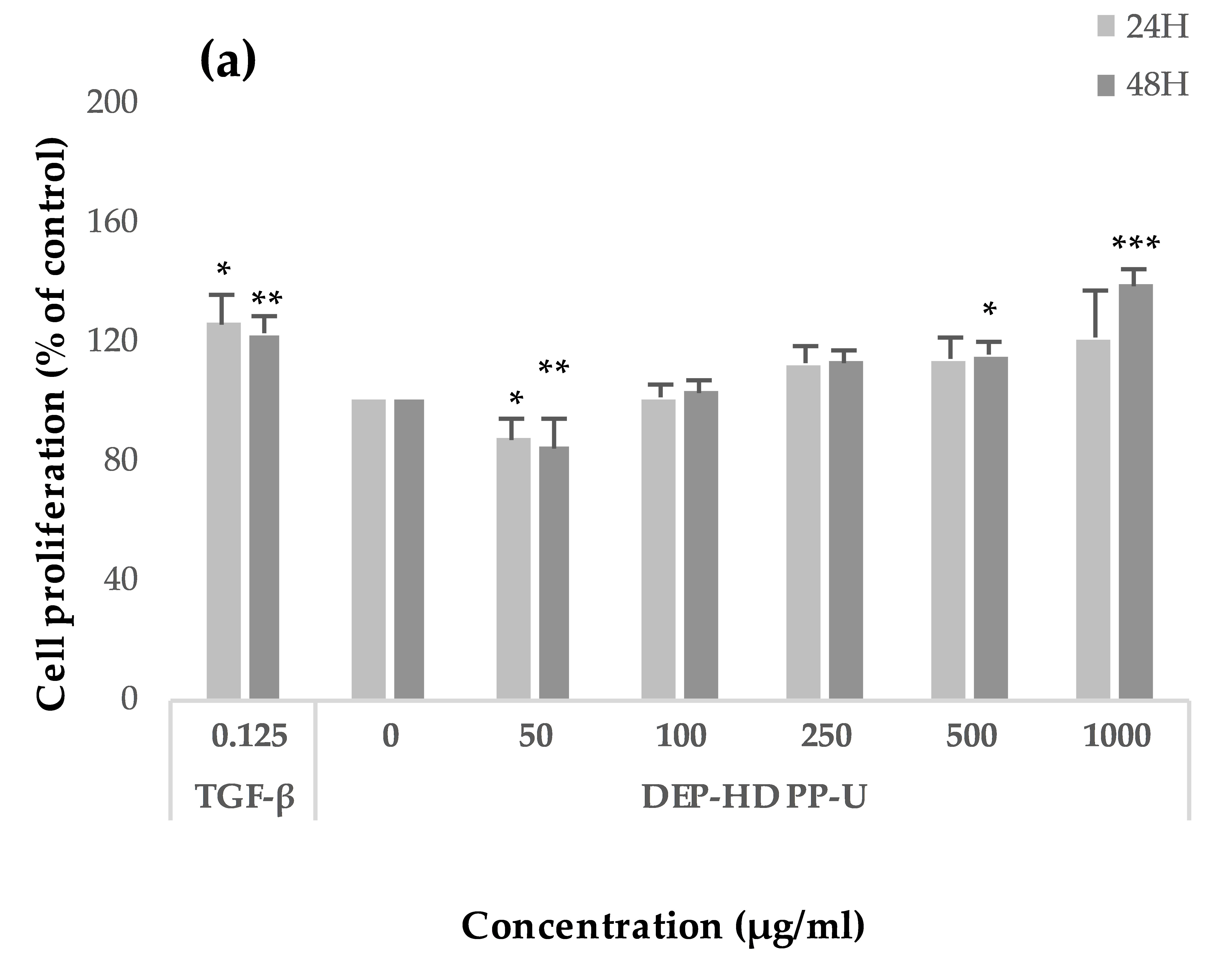

Supplement: Supplementary file 1 [file metabolites-09-00182-s001.zip › Figures and Tables TIF/Supplementary materials/Figure S3a.tif]

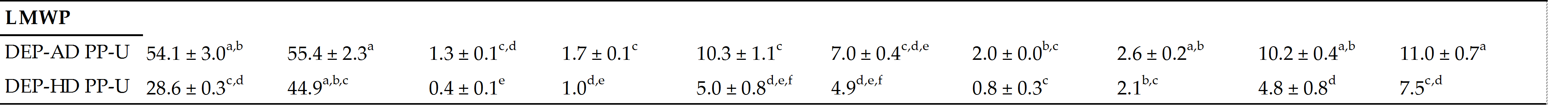

Supplement: Supplementary file 1 [file metabolites-09-00182-s001.zip › Figures and Tables TIF/Supplementary materials/Table S2b.tif]

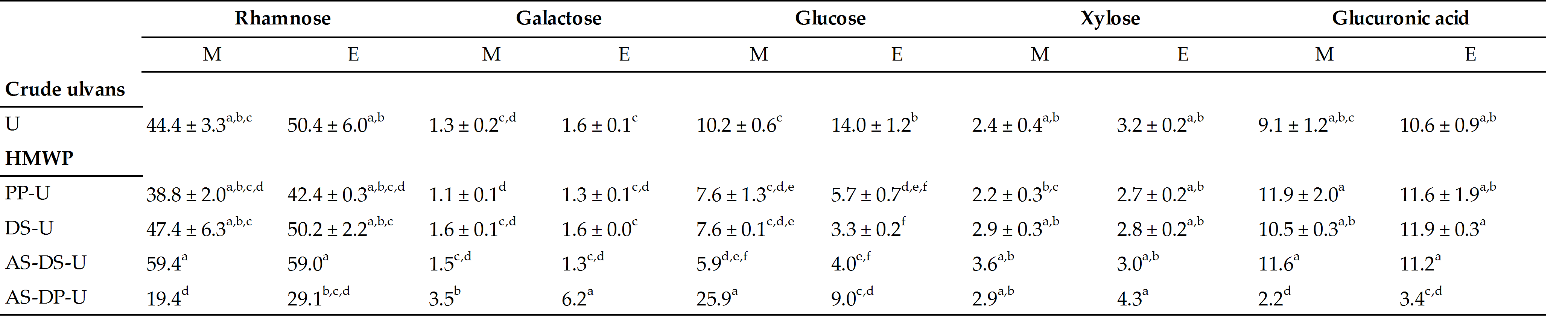

Supplement: Supplementary file 1 [file metabolites-09-00182-s001.zip › Figures and Tables TIF/Supplementary materials/Table S2a.tif]

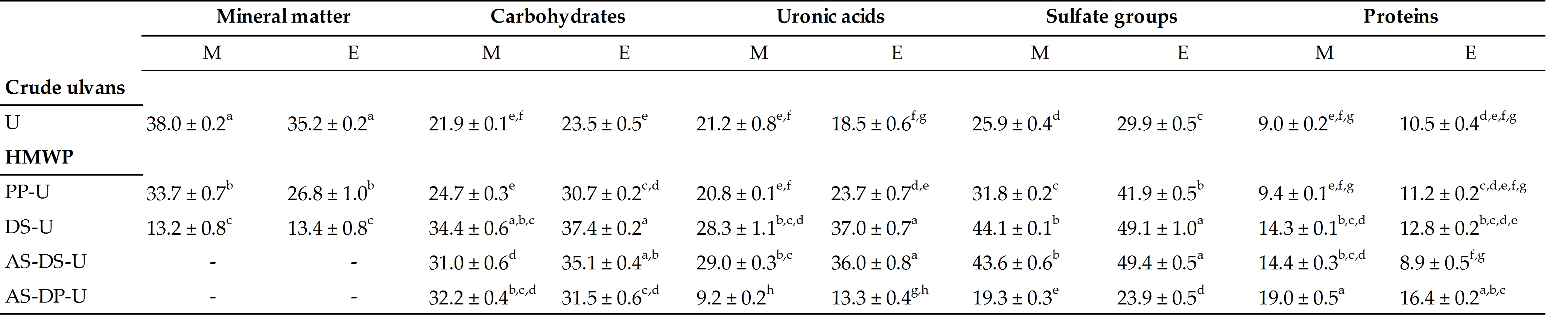

Supplement: Supplementary file 1 [file metabolites-09-00182-s001.zip › Figures and Tables TIF/Supplementary materials/Table S1a.tif]

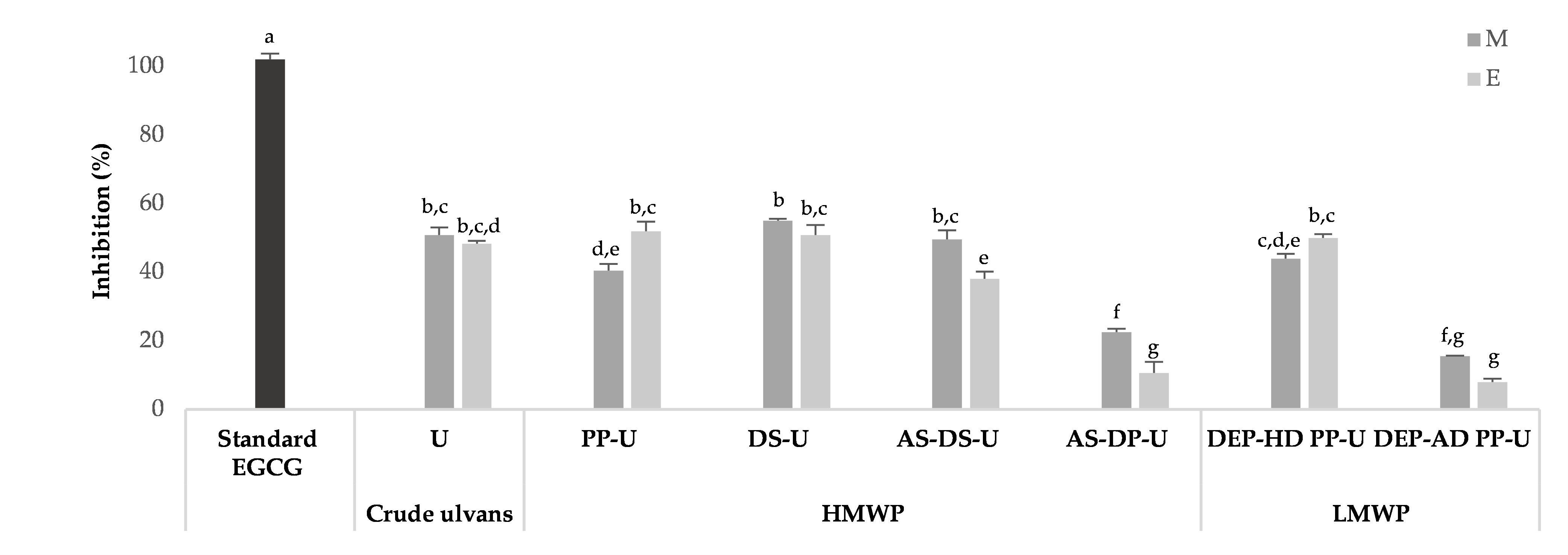

Supplement: Supplementary file 1 [file metabolites-09-00182-s001.zip › Figures and Tables TIF/Supplementary materials/Figure S1.tif]

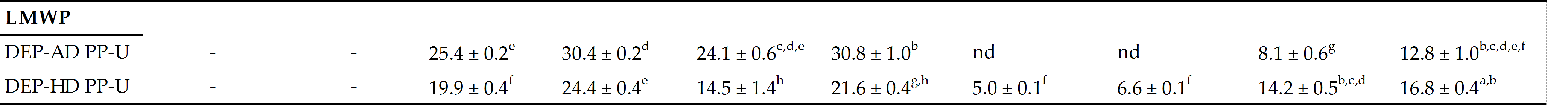

Supplement: Supplementary file 1 [file metabolites-09-00182-s001.zip › Figures and Tables TIF/Supplementary materials/Table S1b.tif]

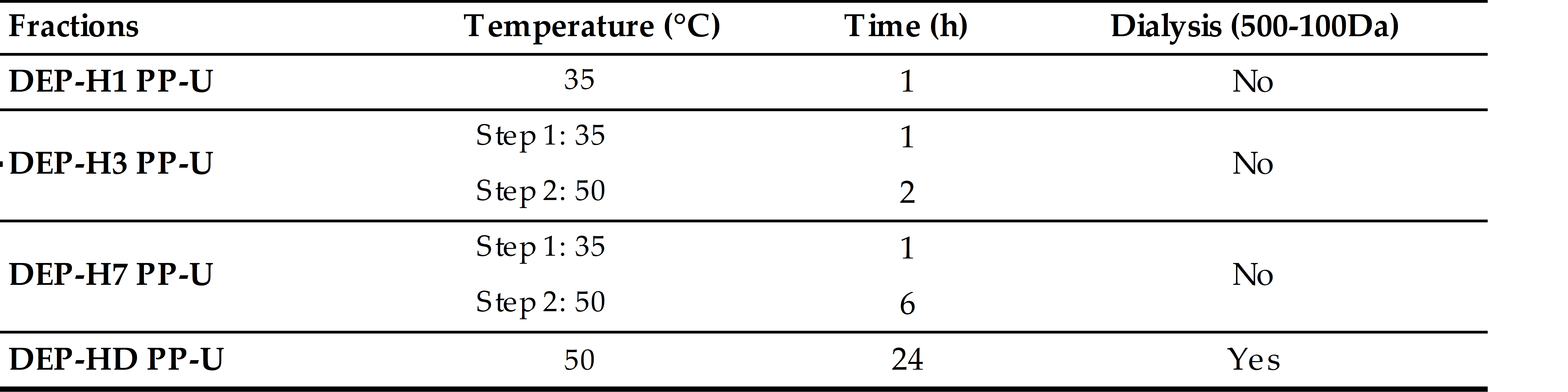

Supplement: Supplementary file 1 [file metabolites-09-00182-s001.zip › Figures and Tables TIF/Table 6.tif]

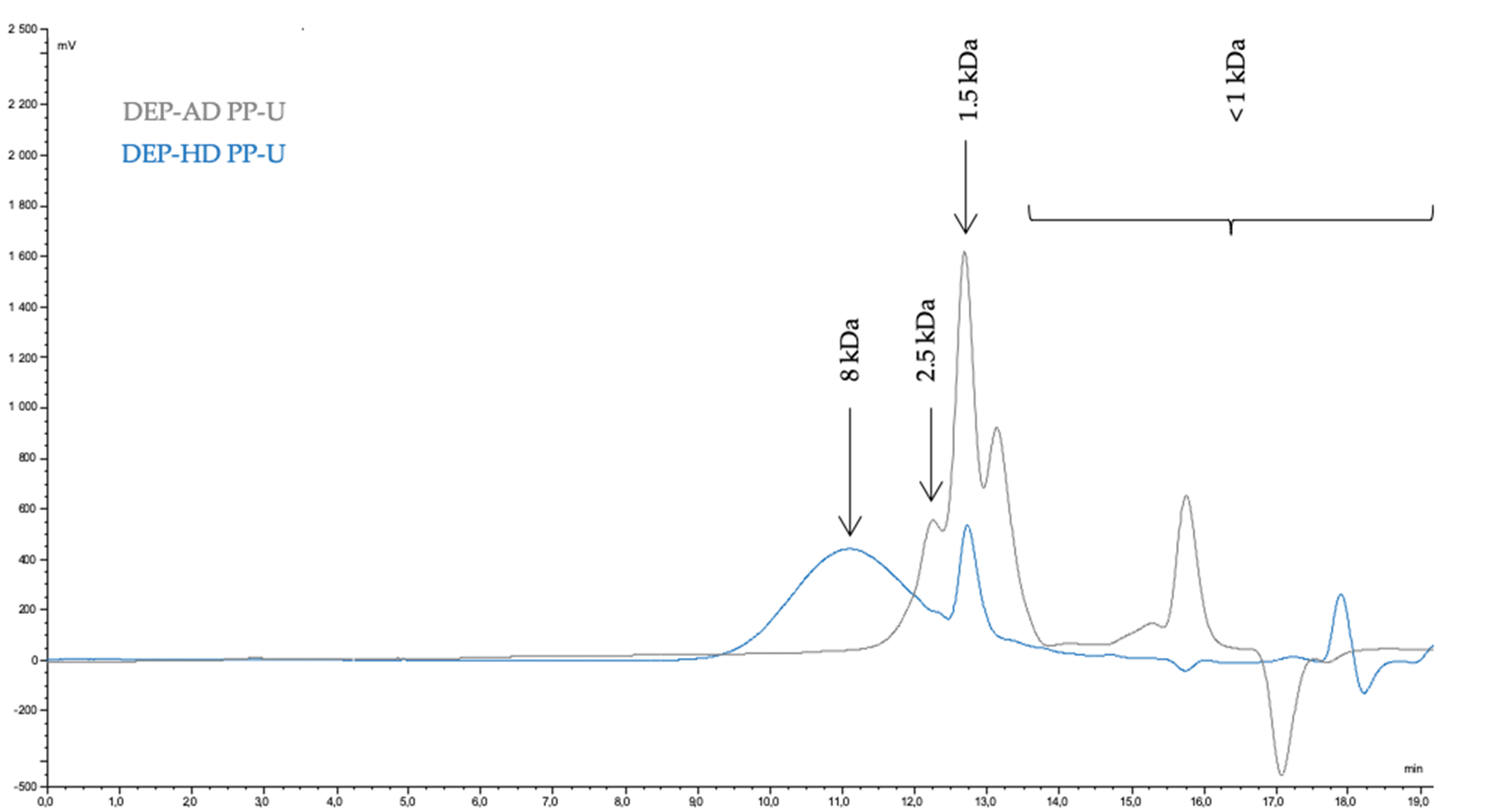

Supplement: Supplementary file 1 [file metabolites-09-00182-s001.zip › Figures and Tables TIF/Figure A1.tif]
